# Sequestration of cucurbitacins from cucumber plants by *Diabrotica balteata* larvae provides little protection against biological control agents

**DOI:** 10.1007/s10340-022-01568-3

**Published:** 2022-10-07

**Authors:** Pamela Bruno, Carla C. M. Arce, Ricardo A. R. Machado, Gaia Besomi, Anna Spescha, Gaétan Glauser, Charlyne Jaccard, Betty Benrey, Ted C. J. Turlings

**Affiliations:** 1 Laboratory of Fundamental and Applied Research in Chemical Ecology, Institute of Biology, University of Neuchâtel, Neuchâtel, Switzerland; 2 Experimental Biology Group, Institute of Biology, University of Neuchâtel, Neuchâtel, Switzerland; 3 Plant Pathology Group, Institute of Integrative Biology, ETH Zürich, Zürich, Switzerland; 4 Neuchâtel Platform of Analytical Chemistry, University of Neuchâtel, Neuchâtel, Switzerland; 5 Laboratory of Evolutionary Entomology, Institute of Biology, University of Neuchâtel, Neuchâtel, Switzerland

**Keywords:** Diabroticina, Biological control, Plant defense, Sequestration, Tritrophic interactions

## Abstract

Cucurbitaceae plants produce cucurbitacins, bitter triterpenoids, to protect themselves against various insects and pathogens. Adult banded cucumber beetles (*Diabrotica balteata*), a common pest of maize and cucurbits, sequester cucurbitacins, presumably as a defensive mechanism against their natural enemies, which might reduce the efficacy of biological control agents. Whether the larvae also sequester and are protected by cucurbitacins is unclear. We profiled cucurbitacin levels in four varieties of cucumber, *Cucumis sativus*, and in larvae fed on these varieties. Then, we evaluated larval growth and resistance against common biocontrol organisms including insect predators, entomopathogenic nematodes, fungi and bacteria. We found considerable qualitative and quantitative differences in the cucurbitacin levels of the four cucumber varieties. While two varieties were fully impaired in their production, the other two accumulated high levels of cucurbitacins. We also observed that *D. balteata* larvae sequester and metabolize cucurbitacins, and although the larvae fed extensively on both belowground and aboveground tissues, the sequestered cucurbitacins were mainly derived from belowground tissues. Cucurbitacins had no detrimental effects on larval performance and, surprisingly, did not provide protection against any of the natural enemies evaluated. Our results show that *D. balteata* larvae can indeed sequester and transform cucurbitacins, but sequestered cucurbitacins do not impact the biocontrol potential of common natural enemies used in biocontrol. Hence, this plant trait should be conserved in plant breeding programs, as it has been demonstrated in previous studies that it can provide protection against plant pathogens and generalist insects.

**Supplementary Information:**

The online version contains supplementary material available at 10.1007/s10340-022-01568-3.

## Key message

- Many herbivorous insects obtain protection against natural enemies by sequestering plant toxins.
- We evaluated whether larvae of *Diabrotica balteata* can sequester cucurbitacins and whether these affect biological control agents.
- Surprisingly, the so-called rootworms fed not only on roots of cucumber plants, but also on stems, cotyledons and leaves.
- However, *Diabrotica balteata* larvae sequestered and metabolized cucurbitacins mainly from roots.
- Cucurbitacins did not affect larval performance and did not protect them against common biological control agents.
- Cucurbitacins sequestered from plant tissues should not affect the biocontrol of *Diabrotica balteata* larvae.

## Introduction

Plants rely on chemical defenses to protect themselves from herbivore attack. In this context, secondary metabolites with toxic, repellent and/or deterrent effects play highly relevant roles (Agrawal et al. 2012; Gershenzon and Dudareva 2007; Steppuhn et al. 2004; Wouters et al. 2016). For the Cucurbitaceae family, which include many species of melons, cucumbers, pumpkins and gourds, the defense against herbivory is primarily mediated by a group of extremely bitter tetracyclic triterpenoids, the cucurbitacins (Chen et al. 2005; Lavie and Glotter 1971; Metcalf et al. 1980; Rehm et al. 1957). These compounds are also present in a few species of other plant families such as Brassicaceae (Curtis and Meade 1971; Nielsen et al. 1977; Sachdev-Gupta et al. 1993), and even in some fungi and bacteria (reviewed in Chen et al. 2005). Cucurbitacins have been shown to be effective in protecting leaves and fruits against attacks by birds, arthropods and pathogens (Bar-Nun and Mayer 1990; Da Costa and Jones 1971; Mason and Turpin 1990; Nielsen et al. 1977; Nishida et al. 1992; Sachdev-Gupta et al. 1993; Tallamy et al. 1997). Cucurbitacins can be highly toxic to vertebrates (David and Vallance 1955), and there are several reports of unfortunate human poisonings (Aldous et al. 1994; Ferguson et al. 1983; Rymal et al. 1984; Verma and Jaiswal 2015), with even a reference of intoxication by gourds written in the Old Testament (“Death in the pot”, 2 Kings 4:38–41, written around the year 600 BC).

Insects have evolved diverse mechanisms to circumvent specific plant defenses, enabling them to optimally exploit their host plants (Ali and Agrawal 2012; Felton and Tumlinson 2008; Zhu‐Salzman et al. 2005). This is also the case for beetles of the subtribe Diabroticina (Coleoptera: Chrysomelidae), which are known to feed on Cucurbitaceae plants despite their toxic cucurbitacins content (Eben 2012; Eben and Barbercheck 1997; Eben et al. 1997; Eben and de Los Monteros 2013; Metcalf et al. 1980). In fact, cucurbitacins even act as strong attractants (kairomones) and phagostimulants for these beetles (Howe et al. 1976; Metcalf et al. 1980). In addition, it has been reported that adults of certain Diabroticina species can sequester and detoxify cucurbitacins, such as the cucurbit specialists *Acalymma vittatum* and few species from the genus *Aulacophora*, as well as the generalist beetles *Diabrotica balteata*, *D. virgifera virgifera*, *D. undecimpunctata*, *D. speciosa* and *Cerotoma arcuata* (Andersen et al. 1988; Ferguson et al. 1985; Nishida et al. 1992). The beetles excrete most of the ingested cucurbitacins, but also conjugate a small proportion that is distributed between the hemolymph, the gut and the rest of the body (Ferguson et al. 1985). This accumulation seems to be persistent (Andersen et al. 1988; Ferguson et al. 1985), and the sequestered cucurbitacins are even transferred to the eggs (Ferguson and Metcalf 1985; Ferguson et al. 1985; Tallamy et al. 1998).

The sequestration of plant secondary metabolites by herbivorous insects often confers protection against natural enemies (Agrawal et al. 2012; Nishida 2002; Opitz and Müller 2009; Robert et al. 2017), a trait that can be linked to their success as pests of many economically important crops and could limit the efficacy of biocontrol strategies (Machado et al. 2020; Robert et al. 2017; Zhang et al. 2019). Ferguson and Metcalf (1985) observed that mantids refused to prey on several beetles belonging to the Diabroticina subtribe, including *Diabrotica balteata* and *Acalymma vittatum*, that had fed on “bitter” squash (assumed to contain cucurbitacins). However, the mantids also rejected a large number of *Acalymma* beetles that had fed on a non-bitter diet. Since all the beetles had fed on bitter squash as larvae, it was concluded that these rejected beetles had sequestered the cucurbitacins at their larval stages. The study by Ferguson and Metcalf (1985) is considered to provide the first evidence of the sequestration of cucurbitacins by Diabroticina beetles and larvae as a defense against natural enemies. However, this protection hypothesis is controversial, as later studies were inconsistent, and did not always find similar effects for Diabroticina beetles (Gould and Massey 1984; Howe et al. 1976; Nishida and Fukami 1990), eggs (Brust and Barbercheck 1992b; Tallamy et al. 1998) and larvae (Barbercheck 1993; Barbercheck et al. 2003, 1995; Eben and Barbercheck 1997). Hence, there is need for more knowledge on the phenomenon and function of cucurbitacin sequestration by Diabroticina larvae.

In this study, we extensively investigated the sequestration of cucurbitacins from cucumber plants (*Cucumis sativus* L) by larvae of the banded cucumber beetle *Diabrotica balteata* LeConte and its effects on common biological agents. To this end, we profiled the main cucurbitacins in different plant organs from four commercial cucumber varieties and analyzed the cucurbitacins accumulated over time by *D. balteata* larvae fed on these plants. To test for costs of the sequestration, we evaluated the weight gain and mortality of larvae that were fed on cucumber plants with or without cucurbitacins. The larvae were found to not only feed on roots, but also extensively on the aboveground plant parts. We therefore assessed whether their performance and the sequestration of cucurbitacins were dependent on which plant organs they had fed on. Subsequently, we determined the mortality of *D. balteata* larvae fed on cucurbitacin-containing and cucurbitacin-free cucumber plants when exposed to insect predators, entomopathogenic nematodes, fungi and bacteria. The results show that *D. balteata* larvae can indeed accumulate considerable amounts of cucurbitacins, mainly from roots, which they convert into other chemical forms. This sequestration was not deleterious to their development and, surprisingly, did not affect the efficacy of biological control agents.

## Materials and methods

### Biological resources

#### Plants

Four commercial varieties of cucumber (*Cucumis sativus* L.) with contrasting cucurbitacin biosynthetic capacities were used for experiments: Tanja (“T”, Zollinger Bio, CH), Sonja F1 EKO (“S”, Koeman Flowerbulbs, NL), Hokus EKO (“H”, Koeman Flowerbulbs, NL) and Marketmore 76 (“M76”, Southern Exposure Seed Exchange, USA). Maize (*Zea mays* L.) seedlings of the hybrid DFI 45,321 (DSP, Delley, CH) were used to rear *D. balteata*. Cucumber plants were grown under phytotron conditions (400 µmol/m^2^/s—LD 16:8—28:26 °C, RH 60%) in potting soil (Gebrüder Patzer GmbH and Co. KG, DE), watered every other day and fertilized twice a week (8–8–6 NPK Maag Garden, CH). The plants used in the experiments were grown either individually or in group of 5 to 10 plants per pot (2 l) to profile cucurbitacins or to feed to larvae in the different experiments, respectively. Experiments were performed with cucumber plants with two expanded leaves (15–20 days). For assays with entomopathogenic bacteria, 3-day-old seedlings of the cucumber varieties were used.

### *Insects, entomopathogenic nematodes, fungi* and *bacteria*

*Diabrotica balteata* eggs were provided by Syngenta Crop Protection (Stein, CH). Because neonate *D. balteata* larvae had extremely high mortality rates on cucumber plants, regardless of the cucurbitacin content in the plants, neonates were first fed on seedlings of maize hybrid DFI 45,321 for 5–7 days and then switched to cucumber plants of the different varieties.

The predators used were adults of the greenhouse rove beetle *Dalotia coriaria* Kraatz (Coleoptera: Staphylinidae), larvae of the common green lacewing *Chrysoperla carnea* Stephens (Neuroptera: Chrysopidae) and adults of the minute pirate bug *Orius laevigatus* Fieber (Heteroptera: Anthocoridae). All the predators were purchased from Andermatt Biocontrol Suisse AG (Grossdietwil, CH) and kept under controlled conditions (LD 16:8–22 °C, RH 60%), and they were fed on *Ephestia kuehniella* eggs (Andermatt Biocontrol Suisse AG, CH) until used for experiments (1–2 days). The *Heterorhabditis bacteriophora and H. zacatecana* entomopathogenic nematodes (EPN) were collected in Mexico as described (Bruno et al. 2020; Machado et al. 2021). The commercial *H. bacteriophora* strain was obtained from e-Nema (Schwentinental, DE). All EPN isolates were reared in last instar *Galleria mellonella* larvae using White traps as previously described and stored at 12 °C in the dark (Bedding and Akhurst 1975; Bruno et al. 2020; Campos-Herrera et al. 2015; White 1927).

The strains of entomopathogenic fungi *Metarhizium brunneum* BIPESCO5 (strain F52, EFSA, 2012; available as “Met52 Granular” by Novozymes Switzerland AG, CH) and *Beauveria bassiana* Naturalis (strain ATCC 74040, EFSA, 2013; available as “Naturalis-L” by Andermatt Biocontrol AG, CH) were provided by Agroscope Reckenholz (Zurich, CH). The entomopathogenic fungi were cultured on larvae of *Galleria mellonella*, re-isolated and cultured on selective Strasser Medium agar at 24 °C in the dark (Strasser et al. 1996). Conidia from the third generation were collected from culturing agar plates by suspending them in 1 ml 0.01% aqueous Tween80 (Sigma-Aldrich, CH). To collect the fungal spores, the resulting conidia suspensions were passed through medical gaze and centrifuged for 10 min at 2,700 g and 15 °C. Supernatants were discarded, and the pellets were re-suspended in distilled water. For experiments, the concentrations were adjusted to 10^3^, 10^5^ and 10^7^ spores per gram of sand. Bacterial strains of *Pseudomonas protegens* CHA0 (Ruffner et al. 2013; Stutz et al. 1986) and *Pseudomonas chlororaphis* PCL1391 (Chin-A-Woeng et al. 1998; Ruffner et al. 2013) were cultured from glycerol stocks at the Plant Pathology Group (ETH Zurich, CH). The bacteria strains from the stocks were streaked with an inoculation loop on King's B (King et al. 1954) agar supplemented with ampicillin (40 µg/ml), cycloheximide (100 µg/ml) and chloramphenicol (13 µg/ml) (KB^+++^) and grown for two days at 24 °C. These colonies were used to inoculate Luria Bertani liquid cultures prepared with Lysogeny broth (LB; 10 g Bacto Tryptone, 5 g Bacto Yeast Extract, 0.25 g MgSO_4_ × 7H_2_O, 8 g NaCl dissolved in 1 L double-distilled H_2_O) (Bertani 1951). The inoculated liquid cultures were incubated overnight at 24 °C and 180 rpm. The cultures were centrifuged at 2700 rpm for 10 min at 4 °C, the supernatants were discarded, and the pellets were re-suspended in NaCl 0.9% by vortexing. For experiments, the concentration was adjusted to 10^8^ cfu/ml (OD_600_ ≈ 0.125).

### Cucurbitacin measurements

To profile plant cucurbitacins, we harvested plants that were grown individually and the roots, stems, cotyledons and leaves of the four plant varieties were separately collected from undamaged plants or plants damaged by 15 second-instar *D. balteata* larvae that had freely fed on them for five days (*n* = 5). Plant organs were flash frozen in liquid nitrogen and ground into a fine powder. To profile insect-sequestered cucurbitacins, second-instar *D. balteata* larvae were fed freely on whole cucumber plants potted for three, five or seven days (*n* = 5–10) or were fed on detached roots or shoots for five days (*n* = 5). The detached plant parts were offered to the larvae by placing them in a plastic pot filled with a thin layer of soil. After feeding, the insects were collected and dissected and their guts were removed to specifically quantify cucurbitacins stored in other insect tissues and hemolymph. Eight to ten dissected larvae were pooled per biological replicate. Insect samples were flash frozen in liquid nitrogen and ground into a fine powder.

To extract cucurbitacins, 100 mg of plant material or 20 mg of larval tissue per sample was suspended in 1 ml or 500 µl of pure MeOH, respectively. Then, the samples were vortexed for 1 min and centrifuged at 20,000 g for 20 min at 4 °C. All the supernatants were filtered (13 mm Syringe filter, PTFE hydrophilic, 0.22 µm, BGB, CH) before analysis by liquid chromatography–mass spectrometry. The quantification of cucurbitacins was performed using a Waters Acquity UPLC™ system equipped with an Acquity UPLC BEH C18 column (Waters, 2.1 × 50 mm, 1.7 μm particle size) and connected to a Synapt G2 QTOF mass spectrometer (Waters, Milford, MA, USA). The entire system was controlled by Masslynx 4.1. The mobile phases consisted of formic acid (0.05%) in water (A) and in acetonitrile (B). A linear gradient from 5–100% B in 7 min was performed, following by washing at 100% B for 2 min and requilibration at 5% B for 2 min. The flow rate was 0.4 ml/min, and the injection volume was 2.5 μl. The mass spectrometer was operated in negative electrospray ionization over a mass range of 85 – 1200 Da. Accurate mass measurements (< 2 ppm) were obtained by infusing a solution of leucine–enkephalin (0.5 μg/ml) throughout the run. Cucurbitacins were putatively identified based on their retention times, exact masses and relative mass defects (allowing for molecular formula determination) (Ekanayaka et al. 2015) and compared with those of the standard Cucurbitacin B as well as with available databases such as the Dictionary of Natural Product (CRC Press). In some cases, the presence of several possible cucurbitacin isomers prevented full identification. Cucurbitacin B solutions at 0.02, 0.08, 0.4, 2, 5 and 10 μg/ml were used to establish a calibration curve for quantitation, and all other cucurbitacins were determined as cucurbitacin B equivalents. Data for quantitation were processed in TargetLynx XS (Waters).

### Performance of *Diabrotica balteata* larvae on cucumber plants

To evaluate the performance of *D. balteata* larvae on the different plant varieties, we fed *D. balteata* larvae on whole plants, on only roots, or only shoots and measured insect weight and mortality. Insects were released in 250-ml plastic pots containing 10 g of moist potting soil (Gebrüder Patzer GmbH & Co. KG, DE) and either roots, shoots or whole plants. Five independent pots with fifteen first-instar larvae per treatment were evaluated (*n* = 5). The detached roots were carefully covered with the soil to avoid desiccation. Pots were maintained in a plant growth chamber (LD 16:8–22 °C, RH 60%). The mean weight and mortality of the larvae were recorded for five days by counting and weighing together the surviving larvae of each sample, and the weight gain per larva was calculated. At the end of the experiment, the surviving larvae were dissected to measure cucurbitacins as described above.

### Bioassays with natural enemies

All experiments with natural enemies were conducted on *D. balteata* larvae previously fed on cucumber varieties (T, S, H and M76, see biological resources) for five days. The experiments were carried out in 30 ml plastic cups with lids (Frontier Scientific Services, Inc., USA) with either moist filter paper on the bottom (predators) or with a 5 mm layer of moist autoclaved washed sand (Ø 1–4 mm) (Migros, CH) (entomopathogenic nematodes, fungi and bacteria).

Predation assays were carried out with *Dalotia coriaria* adults, *Chrysoperla carnea* larvae and *Orius laevigatu*s adults (*n* = 30). One *D. balteata* larva and one predator were released in a 30-ml plastic cup with a moist filter paper on the bottom of the cup. The cups were closed with lids and stored in the dark at 24 °C. The total number of samples with a dead larva that was at least partially eaten by a predator was determined after 48 h. The infectivity of 22 *Heterorhabditis bacteriophora* and three *H. zacatecana* nematode isolates was carried out as described by Zhang et al. (2019) and Bruno et al. (2020) (*n* = 10–20). Approximately 25 newly emerged EPN suspended in 500 µl of tap water were applied to each plastic cup. Each cup contained four *D. balteata* larvae. All cups were closed with lids and kept in the dark at 24 °C. Infection by EPN was verified after 5 days, which was done by evaluating whether the larvae turned orange–red. This color change is due to the endosymbiotic bacteria that grow inside of the host once the nematodes release them (Forst and Clarke 2002) and is depicted in Bruno et al. (2020).

To evaluate EPN reproductive potential, 13 out of the 25 EPN isolates were randomly chosen from the high number of isolates previously tested. For each isolate, four *D. balteata* larvae were infected as described above, but with 125 newly emerged EPN instead of 25 (*n* = 10). Five days after, nematode infectivity was recorded. Five days later, 2 ml of tap water were added to each of the plastic cups that contained at least one infected larva (2–10 cups per variety and EPN strain). Another five days later, this is 15 days after EPN infection, cups were gently shaken, 5 drops of 20 µl were taken from each sample, and the number of infective juveniles (IJs) was counted under a microscope. The average number of IJs counted in the five drops was then used to extrapolate the concentration of IJs in the sample, and this was divided by the number of infected larvae per sample.

Bioassays with entomopathogenic fungi (EPF) were performed with *Metarhizium brunneum* BIPESCO5 and *Beauveria bassiana* Naturalis at 10^3^, 10^5^ and 10^7^ conidia/ml (*n* = 30). One ml of conidia suspension was applied to each plastic cup with moist sand and four *D. balteata* larvae. The cups were closed with lids and stored in the dark at 24 °C. Mortality of the larvae caused by EPF was regularly recorded for ten days.

The mortality of *D. balteata* larvae by entomopathogenic bacteria was assessed with *Pseudomonas protegens* CHA0 and *Pseudomonas chlororaphis* PCL1391. Experiments were carried out in plastic cups with moist sand and four *D. balteata* larvae (*n* = 30). As the bacteria had to be ingested to colonize the midgut of the larvae (Kupferschmied et al. 2013), 3-day-old seedlings of the corresponding cucumber varieties were bathed in 10^8^ cfu/ml bacterial suspensions for 30 min and then placed on the sand in the plastic cups, where they were rapidly eaten by the larvae. The cups were closed with lids and stored in the dark at 24 °C. Mortality of the larvae by EPP was regularly evaluated for seven days.

### Statistical analyses

Statistical tests were carried out with R statistical software (v. 4.0.0; R Development Core Team, 2019) using analysis of variance (ANOVA), followed by residual analyses to verify suitability of distributions of the tested models. Differences in cucurbitacin contents in plants and larvae and the larval weight were analyzed using generalized linear models (GLM) with a Gaussian distribution followed by pairwise comparisons of least squares means (*LSMeans)*. Orthogonal partial least squares discriminant analyses (OPLS-DA) and hierarchical heatmaps were carried out using MetabolAnalyst 5.0 (Chong et al. 2019) to check for differences among cucurbitacins in plant organs and larvae. Larval mortality was compared within each natural enemy for the different food sources using generalized linear models (GLM) under binomial distribution followed by pairwise comparisons of least squares means (*LSMeans)*.

## Results

### *Diabrotica balteata* larvae feed on above and belowground organs of cucumber plants.

**Fig. 1:**
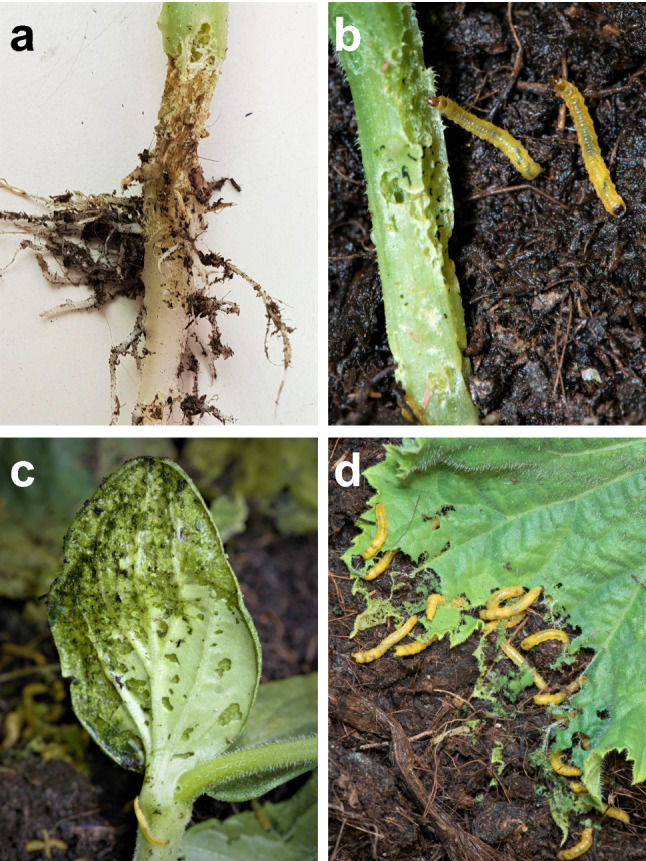
*Diabrotica balteata* larvae feed on above and below ground tissues of cucumber plants. Larvae feeding on **a** roots, **b** stems, **c** cotyledons and **d** leaves. Photographs: © Neil Villard & Pamela Bruno, FARCE

We observed that *Diabrotica balteata* larvae settle mainly on the base of the plants, feeding on the stems, causing plant wilting and stem bending. When the plant leaves are in contact with the soil, the larvae rapidly start feeding on them, but avoid the trichomes (Fig. 1). When the larvae feed on aboveground organs, they become intense yellow, whereas when they only feed on roots, their color is light cream (Fig. S1).

### Cucumber varieties contain different cucurbitacin levels in their organs, and *Diabrotica balteata* larvae sequester cucurbitacins from cucumber plants mainly from the roots.

**Fig. 2:**
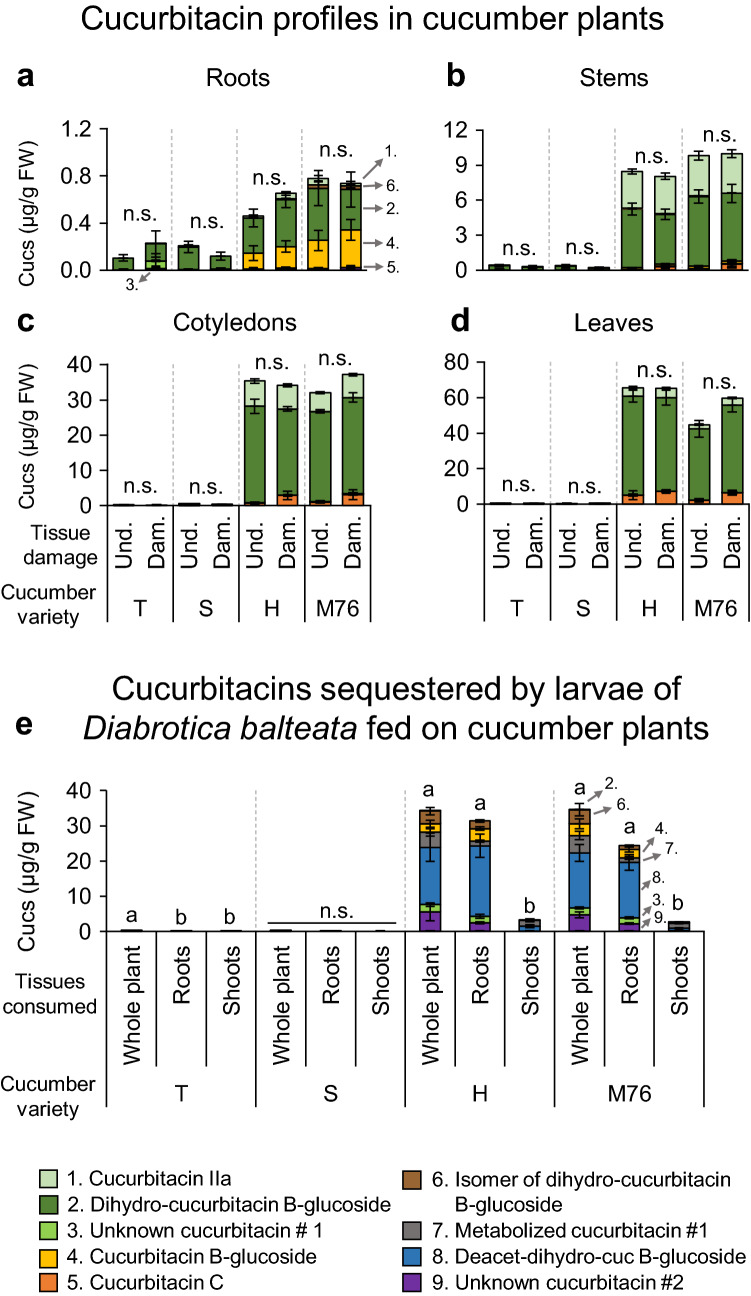
Cucumber varieties have different cucurbitacin levels in their tissues and *Diabrotica balteata* larvae sequester cucurbitacins from cucumber plants, mainly from the roots. (a-d) Cucurbitacins present in cucumber roots (**a**, *n* = 7–8), stems (**b**, *n* = 7–9), cotyledons (**c**, *n* = 7–8) or leaves (**d**, *n* = 7–8) of healthy plants (Undamaged: “Und.”) or after 15 second-instar *D. balteata* larvae freely fed on the plants (Damaged: “Dam.”) for five days. **e** Sequestered cucurbitacins in *D. balteata* larvae after freely feeding for five days on whole cucumber plants or only on roots or shoots (*n* = 5). Commercial varieties of cucumber were used for the experiments: “T” (Tanja), “S” (Sonja), “H” (Hokus) or “M76” (Marketmore 76). Bars indicate average (± SE). *p* values are given for treatments [generalized linear model (family, Gaussian)] followed by pairwise comparisons of least squares means (LSMeans). Not significant (n.s., *p* > 0.05). Different letters indicate significant differences among plant tissues within each cucumber variety, *p* < 0.05. Cucurbitacins present in plant and larvae are indicated with different colors and numbered 1–9 in the bars and in the legend: 1. Cuc IIa, 2. Dihydro-cuc B-glucoside, 3. Unknown cuc#1, 4. Cuc B-glucoside, 5. Cuc C, 6. Isomer of dihydro-cuc B-glucoside, 7. Metabolized cuc #1, 8. Deacetylated-dihydro-cuc B-glucoside, 9. Unknown cuc#2

**Fig. 3:**
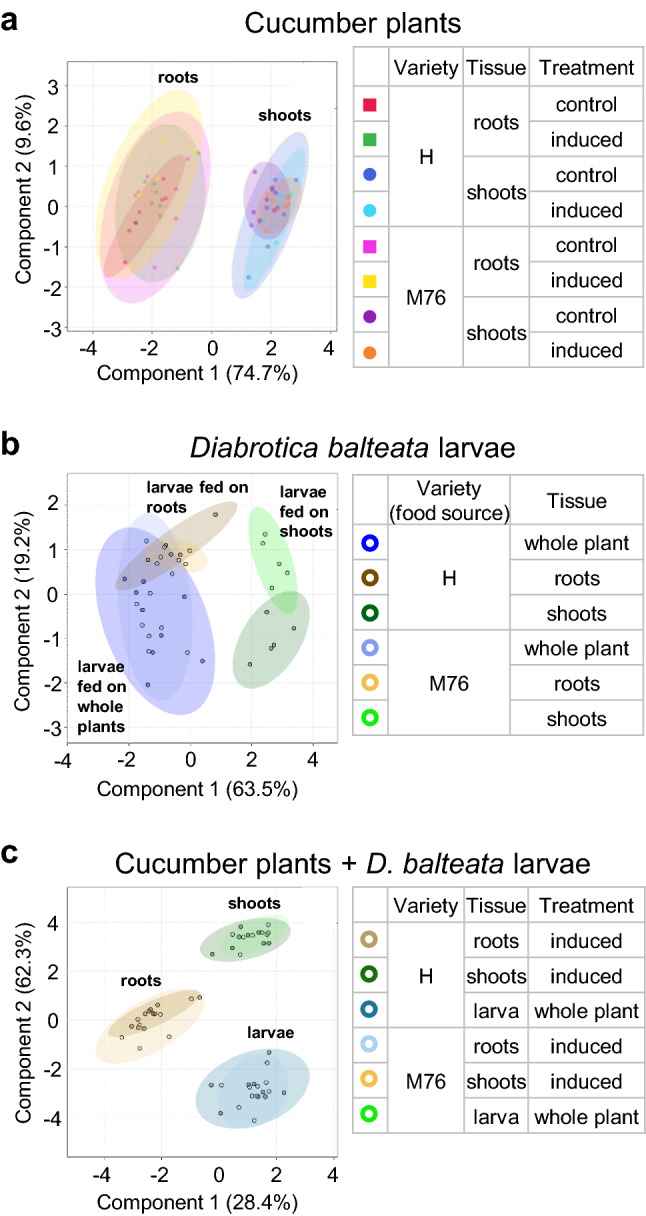
Differences in cucurbitacins in cucumber plants and sequestered by *Diabrotica balteata* larvae fed on those plants. Results of a discriminant analysis (OPLS-DA) of the contents of cucurbitacins in **a** cucumber roots or shoots, either healthy or damaged by *D. balteata* larvae, in **b** *D. balteata* larvae fed on cucumber roots, shoots or whole plants, and **c** comparison of cucurbitacins in cucumber roots or shoots and in *D. balteata* larvae fed on those tissues

Cucurbitacins were mainly found in roots, stems, cotyledons and leaves of cucumber plants from two commercial varieties (Hokus EKO “H” and Marketmore 76 “M76”), and in both undamaged plants and plants damaged for five days by 15 second-instar *D. balteata* larvae (Fig. 2a–d). We found nine main cucurbitacins, which were putatively identified according to their mass spectra after fragmentation (Table S1). The varieties T and S had very low contents of cucurbitacins in all the measured plant organs, with less than 1 µg/g of total cucurbitacins, whereas the cucurbitacin levels for the varieties H and M76 were more than 100 times higher. For H and M76, the cucurbitacin profiles were composed of three metabolites that represented up to 99.5% of the total contents: dihydro-cucurbitacin B-glucoside (77–85%, respectively), cucurbitacin IIa (12–14%, respectively) and cucurbitacin C (4–9%, respectively). For the four varieties, 71 to 99% of the total cucurbitacin contents were found in aboveground organs, and overall, the cucurbitacin contents were very similar for undamaged and damaged plants, suggesting no induction of cucurbitacins (Fig. 2a–d, Fig. 3a, Fig. S2 a). However, stems of H plants had significantly higher levels of cucurbitacin C in damaged plants compared to undamaged plants: 0.04 and 0.31 µg/g, respectively (F_2,8–9_ = 5.2159, *p* = 0.04) (Fig. 2b, Fig. 3a, Fig. S2 a). Although there was no significant difference in the total content of cucurbitacins in the variety M76 after *D. balteata* fed on cotyledons (F_2,8–9_ = 3.40, *p* = 0.08), there was a slight increase in the total value, with 32 µg/g in undamaged and 37 µg/g in damaged cotyledons. This change was mainly due to a significantly higher amount of cucurbitacin IIa in damaged cotyledons (F_2,8_ = 5.6049, *p* = 0.03) (Figs. 2c, 3a, Fig. S2 a). Similarly, for this variety the total quantity of cucurbitacins in the leaves was 45 µg/g in undamaged and 60 µg/g in damaged leaves (F_2,8–9_ = 3.80, *p* = 0.07), which can be explain by significantly higher amounts of cucurbitacin C and cucurbitacin IIa in damaged leaves (cucurbitacin C: F_2,8_ = 5.6623, *p* = 0.03, cucurbitacin IIa: F_2,8_ = 10.0630, *p* = 0.006) (Figs. 2d, 3a, Fig. S2 a).

After *D. balteata* larvae fed for five days on whole plants or only on roots or shoots of the four cucumber varieties, sequestered cucurbitacins were profiled in gut-dissected larvae (Figs. 2e, 3b, c, Fig. S2 b, c). Regardless of the plant organs they had fed on, larvae from T or S plants accumulated negligible amounts of cucurbitacins in their bodies (< 0.3 µg/g). In contrast, larvae fed on whole plants of the varieties H and M76 accumulated up to 34 µg/g of total cucurbitacins and when they were fed only on roots, they accumulated up to 31 µg/g. The profiles of cucurbitacins from larvae that fed either on whole plants or roots were very similar. Surprisingly, the larvae that fed only on shoots sequestered ten times less (up to 3.1 µg/g) (Fig. 2e, Fig. S2 b, c), despite the fact that the leaves contained much higher levels of cucurbitacins than the roots (Fig. 2a–d). The sequestered amount of cucurbitacins in the larvae increased gradually over time after feeding on whole plants of H and M76 for three, five or seven days, and the qualitative composition of cucurbitacins was similar over time (Fig. S3). The similar profiles for cucurbitacins from larvae fed on roots and those fed on whole plants indicate that *D. balteata* larvae accumulate cucurbitacins mainly from the roots (Fig. 3b, c, Fig. S2 b, c). The larvae metabolize the cucurbitacins sequestered from the plants and accumulate transformed types of cucurbitacins in their bodies (Fig. 3b, c). Detailed statistical results are available in Supplementary Tables S2-S7.

### Cucurbitacins from cucumber do not affect the performance of *Diabrotica balteata* larvae

**Fig. 4:**
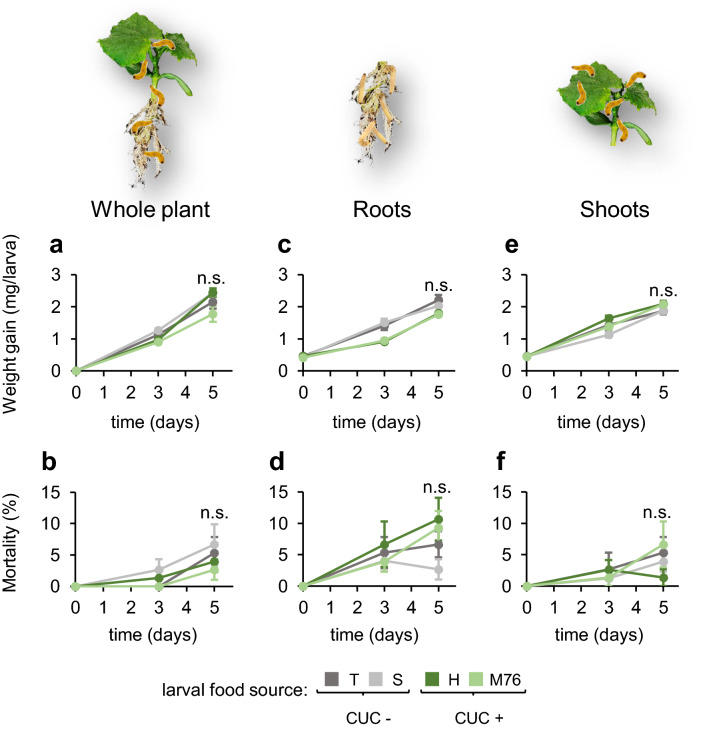
*Diabrotica balteata* larvae do not perform differently on different cucumber varieties. Individual weight gain (mg/larva) and mortality (%) of 15 s-instar *D. balteata* larvae fed on whole cucumber plants (**a**, **b**), or only on roots (**c**, **d**) or shoots (**e**, **f**) for five days (*n* = 5). Commercial varieties of cucumber used for the experiments: “T” (Tanja, dark gray), “S” (Sonja, light gray), “H” (Hokus, dark green) or “M76” (Marketmore 76, light green). Lines indicate average (± SE). Lines indicate average (± SE). P values are given for treatments [generalized linear model (family, Gaussian (weight gain) or binomial (mortality))] followed by pairwise comparisons of least squares means (LSMeans). Not significant (n.s., *p* > 0.05). Photographs: © Neil Villard & Pamela Bruno, FARCE

To determine whether cucurbitacin sequestration influence larval performance, we recorded the mean weight and mortality of *D. balteata* larvae three and five days after they fed on whole cucumber plants or only on roots or shoots of the varieties T, S (CUC -), H or M76 (CUC +), and calculated the weight gain per larva (Fig. 4). After three days of feeding on the different organs and varieties, there were some small differences in the weight gain of the larvae, with higher values for larvae fed on whole plants of S (1.3 mg/larva) compared to the other varieties (0.9 to 1.1 mg/larva) (F_3,3–5_ = 5.35, *p* = 0.009), (Fig. 4 a), as well as for larvae fed on roots of T or S (1 and 1.1 mg/larva, respectively), in comparison with their counterparts fed on H or M76 (0.5 mg/larva) (F_3,3–5_ = 13.90, *p* < 0.001) (Fig. 4 c). In contrast, larvae fed on shoots of S plants had slightly lower weight gain than larvae fed on H plants (0.7 and 1.2 mg/larva, respectively) (F_3,3–5_ = 3.65, *p* = 0.03) (Fig. 4 e). After five days of feeding there were no longer any differences in weight gain for larvae fed on varieties with or without cucurbitacins, regardless of whether they fed on whole plants (F_3,3–5_ = 2.75, *p* = 0.07), or only on roots (F_3,3–5_ = 1.97, *p* = 0.15) or shoots (F_3,3–5_ = 2.93, *p* = 0.06) (Fig. 4a, c, e). Similarly, the larvae fed on the different cucumber varieties showed no differences in mortality after three and five days (Fig. 4b, d, f), regardless of the plant organ consumed and the content of cucurbitacins (after three days, for larvae fed on either entire plants, or just roots or shoots: Chisq_3,5_ = 2.56E-10, *p* = 1.00) (after five days, for larvae fed on whole plants: Chisq_3,5_ = 7.02, *p* = 0.2; for larvae fed on roots: Chisq_3,5_ = 18.03, *p* = 0.85; and for larvae fed on shoots: Chisq_3,5_ = 16.82, *p* = 0.86). These data suggest that cucurbitacins do not affect the performance of *D. balteata* larvae. Detailed statistical results are available in Supplementary Tables S8-S9.

### Cucurbitacins do not protect *Diabrotica balteata* larvae against common natural enemies

**Fig. 5:**
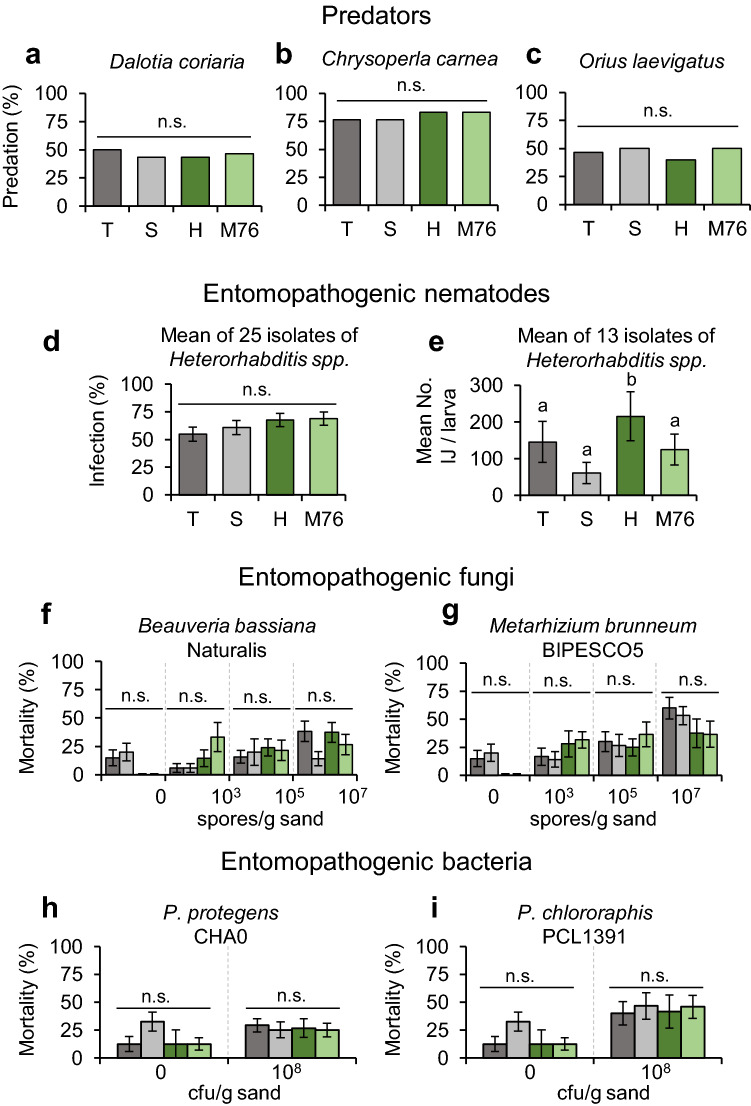
Cucurbitacins do not protect *Diabrotica balteata* larvae against natural enemies. Second-instar *D. balteata* larvae fed for five days on cucumber plants of the commercial varieties “T” or “S” or “H” or “M76”, and then were exposed to natural enemies. **a**–**c** Predation (%) of one *D. balteata* larva 48 h after being exposed to one **a** *Dalotia coriaria* adult, **b** *Chrysoperla carnea* larva or **c** *Orius laevigatus* adult (*n* = 30). **d** Mean infection rates (%) of 22 *Heterorhabditis bacteriophora* and *3 H. zacatecana* isolates five days after inoculation on four *D. balteata* larvae (*n* = 15–20). **e** Mean number of infected juveniles produced by 13 isolates of *H. bacteriophora* and *H. zacatecana* (*n* = 2–10). Mortality of four *D. balteata* larvae seven days after infection with *B. bassiana* (**f**, *n* = 30), *M. brunneum* (**g**, *n* = 30), or five days after infection with *P. protegens* (**h**, *n* = 30) and *P. chlororaphis* (**i**, *n* = 30). Bars indicate percentage (**a**, **b**, **c**, **f**, **g**, **h**, **i**) or average (**d** and **e**) (± SE). *p* values are given for treatments [generalized linear model (family, binomial)] followed by pairwise comparisons of least squares means (LSMeans). Not significant (*n*.s., *p* > 0.05). Different letters indicate significant differences among plant varieties, *p* < 0.05

In order to assess whether sequestered cucurbitacins protect *D. balteata* larvae against natural enemies, larvae fed for five days on whole plants of cucumber varieties with or without cucurbitacins (CUC -: T, S, CUC + : H, M76) were exposed to insect predators, entomopathogenic nematodes (EPN), fungi and bacteria (Fig. 5). The cucurbitacin content in the larvae did not appear to have any effect on the predation rates after 48 h by *Dalotia coriaria* (F_4,30_ = 0.1189, *p* = 0.94) (Fig. 5a), *Chrysoperla carnea* (Chisq = 119.26, df = 26, *p* = 0.84) (Fig. 5b) or *Orius laevigatus* (F_4,30_ = 0.2603, *p* = 0.85) (Fig. 5c). Similarly, the mean infection percentage of 25 EPN isolates on the larvae was not affected by any of the cucumber varieties that they had fed on (Chisq = 37.941, df = 96, *p* = 0.24) (Fig. 5d). Out of the 25 EPN isolates evaluated, only six showed significant differences in infection rates among treatments (Fig. S4). However, the differences cannot be explained by cucurbitacin content; for some isolates infectivity was actually higher in larvae fed on one of the varieties containing cucurbitacins, whereas for other isolates it was the other way around (Fig. S4, Table S11). In order to determine whether the EPN were indirectly affected by the cucurbitacins sequestered by their host larvae, we compared the progeny production of 13 of the 25 isolates previously evaluated (Fig. 5e, Fig. S5). The mean progeny production of the 13 isolates was significantly higher for EPN that had infected larvae fed on cucumber plants of the variety H, compared to the other three varieties (F_4,2–10_ = 8.8908, *p* < 0.001) (Fig. 5e). This was in particular the case of two of the EPN isolates; MEX-21 and MEX-23, whereas for all the other isolates we did not observe differences in the number of infective juveniles (IJs) produced by the EPN that had killed larvae fed on varieties with or without cucurbitacins (Fig. S5). The mortality of *D. balteata* larvae seven days after inoculation with different concentrations of the entomopathogenic fungi *Beauveria bassiana* Naturalis and *Metarhizium brunneum* BIPESCO5 did not differ among the larvae fed on different cucumber varieties (Fig. 5f, g). The mortality of the larvae was generally very low and rapidly increased after seven days, especially for higher fungal concentrations, but this was unrelated to the cucumber varieties that the larvae had consumed (Fig. S6). Similarly, the mortality of *D. balteata* larvae five days after ingestion of entomopathogenic bacteria *Pseudomonas protegens* CHA0 or *P. chlororaphis* PCL 1391 was not significantly different for the four larval food sources (Fig. 5 h, i). In general, the mortality increased after seven days for all the larvae, but this was not consistent for all varieties with or without cucurbitacins (Fig. S7). Overall, cucurbitacins sequestered by *D. balteata* larvae did not provide any protection against the insect predators, EPN, fungi and bacteria evaluated. Detailed statistical results are available in Supplementary Tables S10-S14.

## Discussion

In this study, we could show that *Diabrotica balteata* larvae are able to sequester cucurbitacins. We profiled the cucurbitacins in plants of four commercial cucumber varieties and found that some varieties contain up to 27 µg/g in total contents of cucurbitacins, whereas others have less than 1 µg/g. For cucurbitacin-containing varieties, more than 99% of the cucurbitacins are accumulated in the shoots, in particular in the leaves. The cucurbitacins from cucumber plants showed only minor induction after *D. balteata* larvae fed on the plants for five days. Although *D. balteata* larvae were found to feed extensively on aboveground cucumber organs, they sequestered and accumulated the cucurbitacins mainly from roots, transforming some of them into different cucurbitacins. Cucurbitacin content in the host plants did not exert a measurable effect on larval performance and provided no protection against the evaluated insect predators, entomopathogenic nematodes, fungi and bacteria.

Diabroticina larvae are called rootworms, in reference to their soil-dwelling behavior. We had therefore expected the larvae to only feed on roots, and we were surprised to observe that most of the larvae foraged on the soil surface around the stem base, and that they extensively fed on aboveground organs, while hiding under the leaves. This behavior is not commonly reported, but has been observed for Diabroticina larvae feeding on maize (Canales Santos 1990), beans (Cardona et al. 1982), cucurbits (Jiménez Martínez and Rodríguez Flores 2014) and chia (Sosa-Baldivia and Ruiz-Ibarra 2016). Related to this behavior, we observed that when *D. balteata* larvae fed on aboveground organs, their light cream color changed to an intense yellow, which does not happen when they feed only on roots. Larval color changes after feeding on different plant parts has already been observed in Lepidoptera (Greene 1989; Yamasaki et al. 2009) and Diptera (Zettler et al. 1998), and has been suggested to provide camouflage against predators (Eacock et al. 2017). Whether the color change provides protection to *D. balteata* larvae remains to be tested.

The biosynthesis of cucurbitacins in cucumber is driven by a qualitative gene Bi (bitter foliage) and also by a “bitter fruit” gene Bt dependent on Bi dominance (Andeweg and De Bruyn 1959; Pierce and Wehner 1990). However, abiotic stresses such as drought and heat can increase bitterness (Andeweg and De Bruyn 1959; Kano and Goto 2000), and more genes seem to be involved in the biosynthesis and regulation of cucurbitacins (Shang et al. 2014). Nevertheless, it has been traditionally postulated that cucurbitaceous plants are either entirely “bitter” (with cucurbitacins), or they are “sweet” or “non-bitter” (without cucurbitacins), and that the taste of cotyledons (or even insects that fed on them) can be correlated with the presence or absence of cucurbitacins in the rest of the plant (Agrawal et al. 1999; Brust and Barbercheck 1992a; Deheer and Tallamy 1991; Dhillon 1993; Howe et al. 1976). Our analyses showed that cucurbitacins are distributed differently among the plant organs, which would suggest that plants could not be exclusively classified as “bitter” or “sweet”. In addition, it remains unclear whether the glycosylation of cucurbitacins reduces their bitterness and, in consequence, their toxicity. Although all cucurbitacins are thought to be extremely bitter and to confer toxicity (Chen et al. 2005), the quantitative variation in bitterness and toxicity between aglucones and glucosylated cucurbitacins has only recently been explored (Zhong et al. 2017), and further studies are necessary. As the chemical identities of many of the compounds found in our analyses (including several cucurbitacins that were glucosylated) have not been reported before, no pure standards are available and their bitterness and individual toxicity remain unknown.

We found hardly any induction of cucurbitacins after plants were damaged by larval feeding. Contents in undamaged and damaged organs were generally very similar, with the exception of cucurbitacin C levels in stems of the variety H and leaves of M76, and cucurbitacin IIa glucoside in cotyledons and leaves of M76 cucumber plants, which were higher in damaged plants. Agrawal et al. (1999) had previously reported the induction of cucurbitacin C in the cucumber variety M76, after spider mites fed on the leaves. Similarly, Tallamy (1985) observed an induction of cucurbitacin B and D in mechanically damaged squash leaves. On the other hand, Apriyanto and Potter (1990) did not find an induction of cucurbitacin production on cucumber leaves after inoculation with tobacco necrosis virus (TNV). Induction may therefore depend on the type of attacker and/or be different for different plant species and varieties. It is also plausible that the induction of other defensive metabolites could be triggered in cucumber tissues upon larval feeding.

Other possible effects of induced resistance might include changes in trichomes, such as trichome density and induction of toxic compounds in the trichome cells (Pullin and Gilbert 1989). The trichomes in cucumber are multicellular and contain several secondary metabolites, such as flavonoids (Pan et al. 2021). All the cucumber varieties tested in our experiments had large trichomes on their stems, leaves (mostly on the nerves) and cotyledons. We observed that the larvae clearly avoided the trichomes, feeding around the leaf nerves and on the under sides of leaves and cotyledons, where the trichomes are smaller. It appears that in cucumber plants the trichome density does not change after leaf herbivory (Agrawal et al. 1999). Whether flavonoids or other secondary metabolites in the trichomes are inducible, though, has not yet been tested.

The sequestration and metabolization of cucurbitacins by Diabroticina have been previously studied for adult beetles that either only consumed purified aglucone cucurbitacin B (Ferguson et al. 1985) or fed on squash fruits known to have the (aglucone) cucurbitacins B and D (Andersen et al. 1988). In these experiments, since the beetles only supposedly ingested aglucone cucurbitacins, it was assumed that the insects had hydrogenated, acetylated, desaturated and glucosylated the cucurbitacins in their bodies (Ferguson et al. 1985). Our analyses found hydrogenated, deacetylated and glucosylated cucurbitacins not only in larvae but also in plants. Therefore, we cannot assume, as previously suggested, that these molecules are stable products that result from detoxification of the cucurbitacins ingested by *Diabrotica* beetles (Tallamy et al. 2000). Regarding sequestration in particular, Barbercheck et al. (1995) and Tallamy et al. (1998) quantified cucurbitacins in entire bodies of *D. undecimpunctata howardi* larvae fed on squash. Their results strongly indicated sequestration, but the quantified cucurbitacins in those analyses could also have been from the plant tissues in the larval guts at the moment of quantification. By dissecting the larval guts before the analyses, we could conclusively demonstrate the sequestration of these compounds by *D. balteata* larvae and their accumulation in the non-gut tissues.

Although the highest amount of cucurbitacins was found in aboveground plant organs (in particular for the varieties H and M76), we surprisingly found that larvae accumulated and metabolized cucurbitacins mostly when they fed on roots. Apart from this and the different coloration of the larvae, we did not detect other differences between larvae fed on below versus aboveground organs. The larvae did not obtain an evident nutritional advantage after feeding on roots or shoots, as the weight gain and mortality were very similar for all organs (and varieties). However, we did not analyze the primary metabolism of the plants used in the experiments, and other nutritional aspects of *D. balteata* larvae fed on roots versus shoots might be different, which might help explain why they feed on aboveground organs.

Whether cucurbitacins protect Diabroticina species against natural enemies has been a controversial subject (Tallamy and Krischik 1989). Adult beetles of *D. undecimpunctata* fed on cucurbits have been shown to be rejected by mantids (Ferguson and Metcalf 1985), but not by birds, mice and frogs (Gould and Massey 1984). Eggs from beetles fed on bitter or non-bitter cucumber are equally predated by carabid larvae, mites or centipedes (Brust and Barbercheck 1992a). It is less clear whether there are any effects on entomopathogenic nematodes (Barbercheck et al. 1995) and entomopathogenic fungi (Tallamy et al. 1998). Our results imply that there is indeed no deterrent effect of sequestered cucurbitacins on common natural enemies. A potential limitation of our experimental setup is that we used no-choice experiments to test the effects of cucurbitacins on the predators *Dalotia coriaria, Chrysoperla carnea* and *Orius laevigatus*. It is possible that no-choice tests are less sensitive to detect predator feeding preferences than choice experiments, as hungry predators might prey even on cucurbitacin-containing larvae if no other food is available. It should be noted, however, that, in nature, the predators are unlikely to be confronted with choice situations either. Also, we have recently observed that in choice experiments *D. coriaria* feeds equally on cucurbitacin-free and cucurbitacin-containing larvae (Jaccard et al. 2022), which further confirms that cucurbitacins do not affect the feeding preference of this predator species.

In addition to assessing the direct effects of cucurbitacins sequestered by the larvae on their vulnerability to natural enemies, we also evaluated possible long-term effects on the fitness of one of their main natural enemies, entomopathogenic nematodes. For this, we compared the progeny of the entomopathogenic nematodes that succeeded in killing larvae that had fed on cucumber plants with or without cucurbitacins. Although we observed differences in the number of infective juveniles (IJs) among treatments, these differences did not correlate with the cucurbitacin contents in the plants (Fig. S5). As it has been previously hypothesized, the observed variations could be related to the nutritional quality of the larvae as food sources for the nematodes (Barbercheck et al. 1995). All of our larvae had similar weights after feeding on cucumber plants, but other aspects of larval suitability for nematodes could have been influenced by feeding on the different varieties. We could not assess the survival and performance of predators fed on *D. balteata* larvae with sequestered cucurbitacins due to the reflex bleeding of the larvae. When threatened or attacked, some insects exude hemolymph, which quickly coagulates and can entangle their aggressor and glue the mouthparts of predators together. This so-called reflex bleeding is a defense response in various insects (Lundgren et al. 2009; Wallace and Blum 1971). In our attempts to evaluate survival, all predators eventually died unable to clean themselves despite vigorous attempts. This might suggest that against some natural enemies, this defense strategy could be prevalent for *Diabrotica* larvae to the sequestration of cucurbitacins.

Our results provide first conclusive evidence for the ability of *D. balteata* larvae to sequester cucurbitacins from their host plants, but this does not affect the efficacy of several common biological control agents. The presence of cucurbitacins, which represent a key element of the plants’ defense against pathogens and generalist insects, should therefore not be eliminated from plant breeding programs.

## Author contributions

PB, CCMA, RARM, BB and TCJT conceived the original project. PB, CCMA, RARM, GB, AS, GG and CJ performed experiments. PB and CCMA carried out statistical analyses. PB and CCMA wrote the first draft of the manuscript. All authors contributed to the final manuscript.

## Supplementary Information

Below is the link to the electronic supplementary material.

Supplementary file1 (PDF 1384 KB)

## Data Availability

The data supporting the findings of this study are available in the supplementary materials.
