# Supplementary material for: Sequestration of cucurbitacins from cucumber plants by Diabrotica balteata larvae provides little protection against biological control agents
Source: J Pest Sci (2004). 2022 Oct 7;96(3):1061–75. doi: 10.1007/s10340-022-01568-3 (PMC10169900; doi:10.1007/s10340-022-01568-3)
Supplement: Supplementary file 1 — Supplementary file1 (PDF 1384 KB) [file 10340_2022_1568_MOESM1_ESM.pdf]

Supplementary Results

Sequestration of cucurbitacins from cucumber plants by *Diabrotica balteata* larvae provides little protection against biological control agents

Pamela Bruno[1], Carla CM Arce[1]*, Ricardo AR Machado[2], Gaia Besomi[1], Anna Spescha[3], Gaétan Glauser[4], Charlyne Jaccard[5], Betty Benrey[5] & Ted CJ Turlings[1]*

1. *Laboratory of Fundamental and Applied Research in Chemical Ecology, Institute of Biology, University of Neuchâtel, Switzerland.* https://orcid.org/0000-0002-0595-9478
2. *Experimental Biology group, Institute of Biology, University of Neuchâtel, Switzerland.*
3. *Plant Pathology Group, Institute of Integrative Biology, ETH Zürich, Switzerland.*
4. *Neuchâtel Platform of Analytical Chemistry, University of Neuchâtel, Switzerland*
5. *Laboratory of Evolutionary Entomology, Institute of Biology, University of Neuchâtel, Switzerland.*

**Corresponding authors:* *carla.marques@unine.ch* & *ted.turlings@unine.ch*

ORCID:
Pamela Bruno 0000-0002-0595-9478
Carla CM Arce 0000-0002-1713-6970
Ricardo AR Machado 0000-0002-7624-1105
Anna Spescha 0000-0002-9184-2937
Gaétan Glauser 0000-0002-0983-8614
Charlyne Jaccard 0000-0002-8493-1704
Betty Benrey 0000-0002-3230-4450
Ted CJ Turlings 0000-0002-8315-785X

Journal of Pest Science

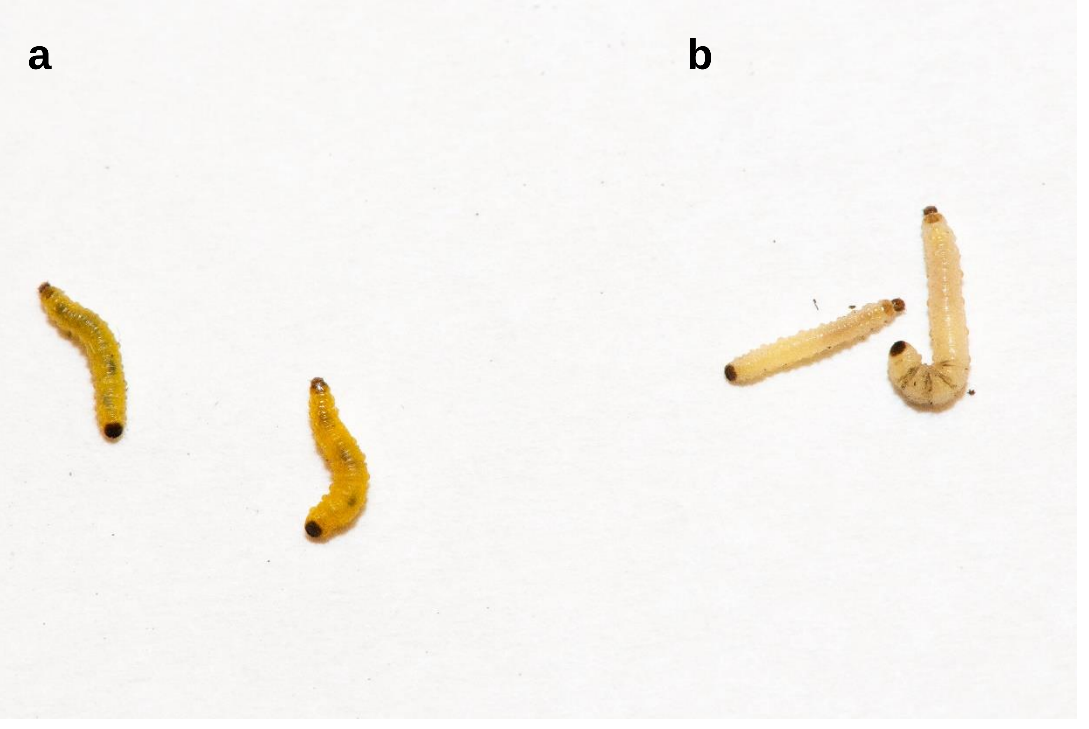

Fig. S1 ***Diabrotica balteata*** **larvae change color when fed on aboveground tissues.**
Third-instar larvae of *D. balteata* after feeding on (**a**) shoots or (**b**) roots of cucumber plants for five days. Larvae fed on shoots are dark yellow, whereas larvae fed only on roots stay light cream. Photos: © Neil Villard & Pamela Bruno, FARCE

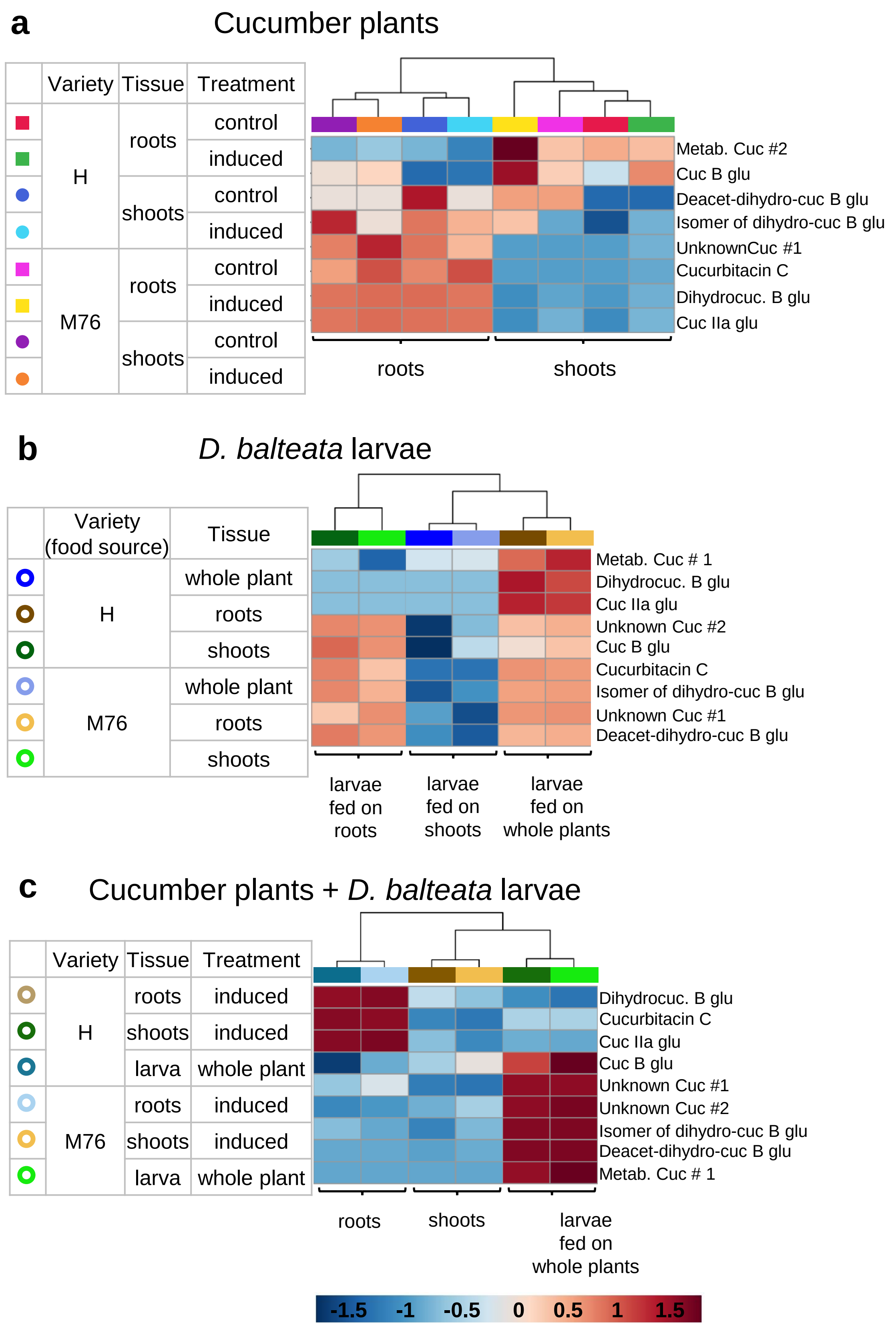

Fig. S2 Differences in cucurbitacins in cucumber plants and sequestered by *Diabrotica balteata* larvae fed on those plants.

Hierarchical clustering heatmaps of the contents of cucurbitacins in (**a**) cucumber roots or shoots damaged by *D. balteata* larvae, in (**b**) *D. balteata* larvae fed on cucumber roots, shoots or whole plants, and (**c**) comparison of cucurbitacins in cucumber roots or shoots and in *D. balteata* larvae fed on those tissues

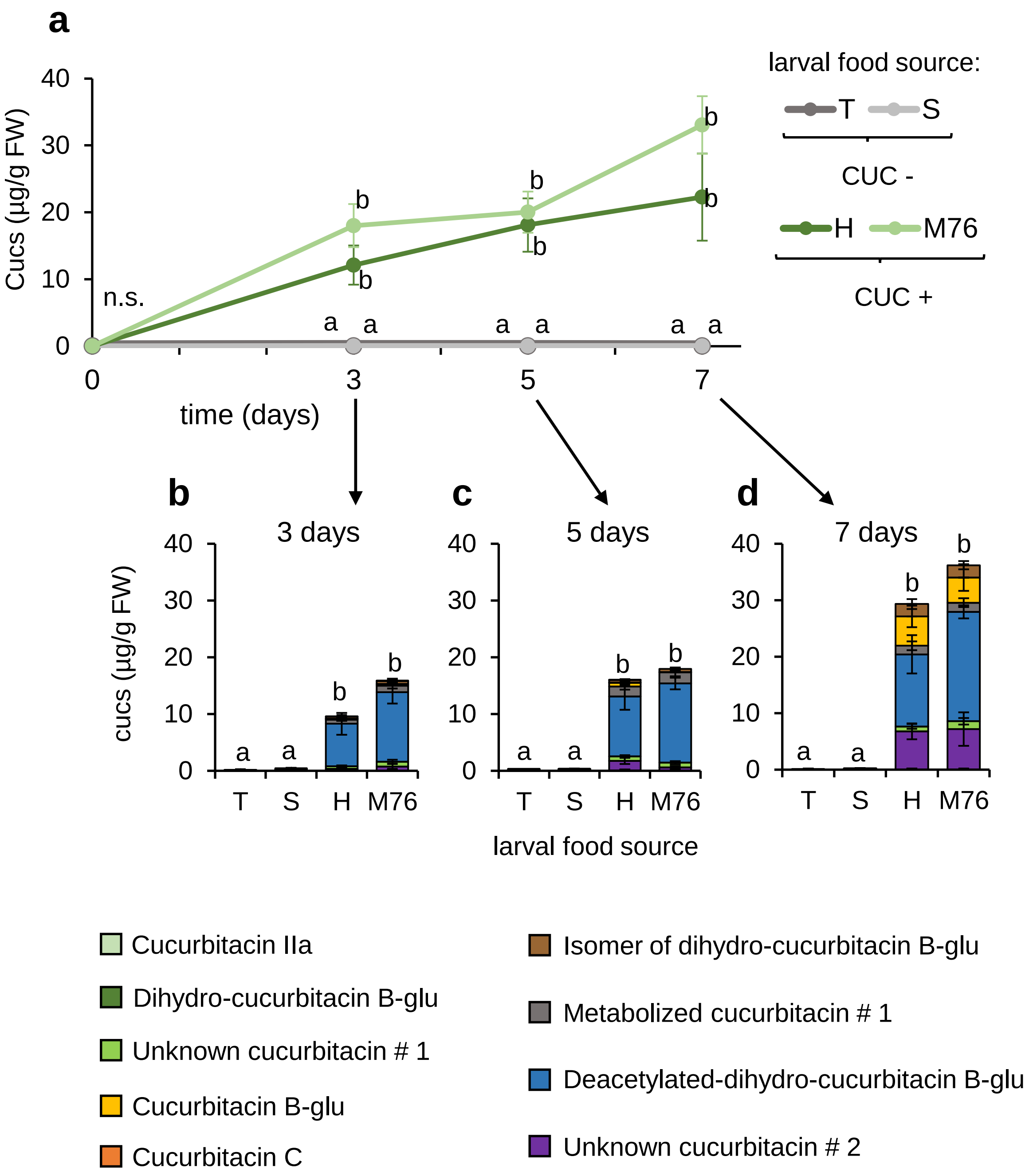

Fig. S3 The accumulation of sequestered cucurbitacins by *Diabrotica balteata* larvae increases over time. Total sequestered cucurbitacins (**a**) and main cucurbitacins (**b**-**d**) in gut-dissected *D. balteata* larvae after freely feeding on cucumber plants of the commercial varieties "T" (Tanja), "S" (Sonja), "H" (Hokus) or "M76" (Marketmore 76) for three (**b**, n=5-9), five (**c**, 7=10) or seven days (**d**, n=5). Bars indicate average (± SE). *P* values are given for treatments [generalized linear model (family, Gaussian)] followed by pairwise comparisons of Least Squares Means (LSMeans). Different letters indicate significant differences among plant tissues within each cucumber variety, p<0.05

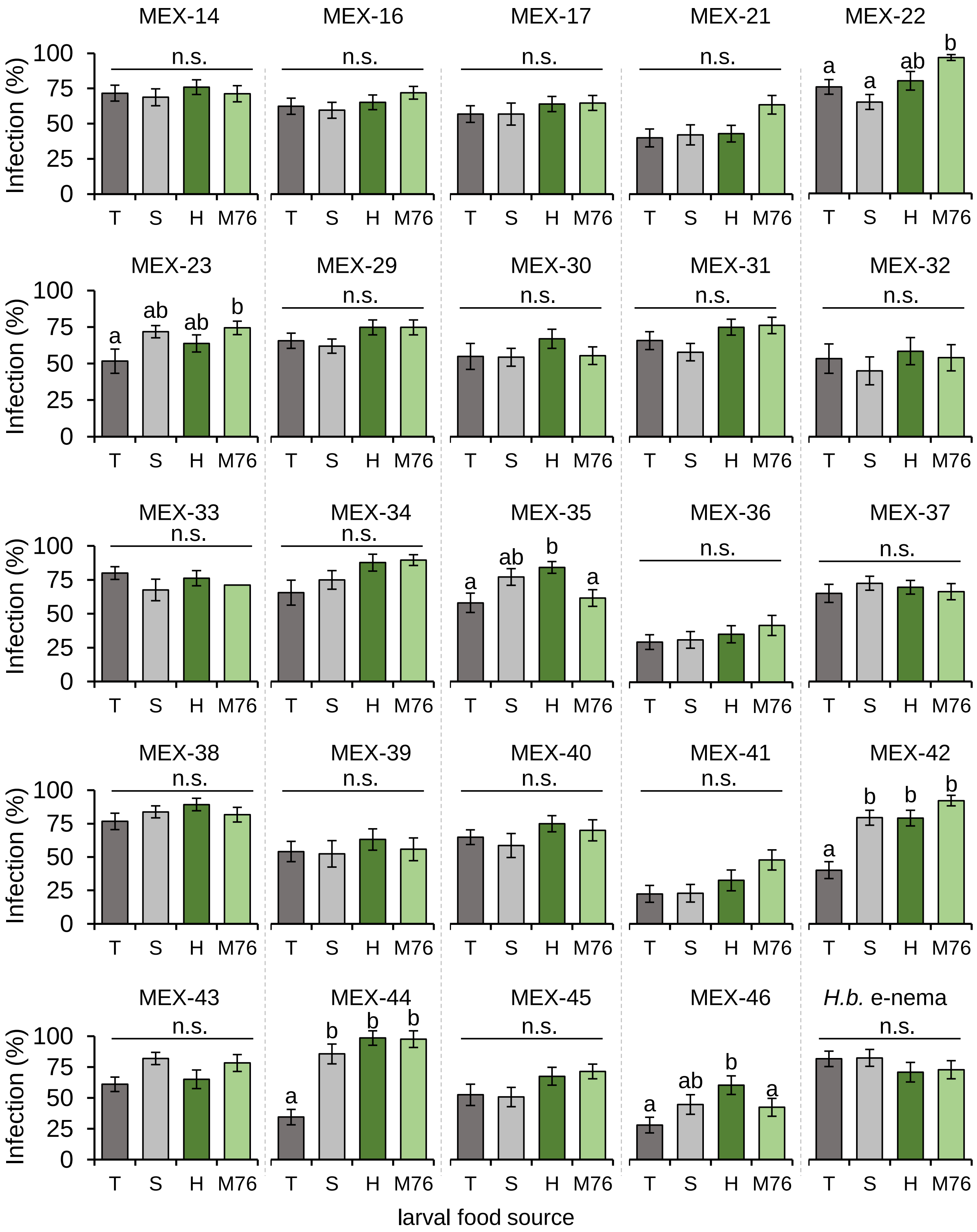

Fig. S4 Cucurbitacins do not protect *Diabrotica balteata* larvae against several isolates of *Heterorhabditis* sp. entomopathogenic nematodes. Mean infection rates (%) of 25 isolates of *Heterorhabditis* sp. entomopathogenic nematodes five days after inoculation with 500 ml *H. bacteriophora* (MEX-14 to MEX-38 and MEX-42 to MEX-46) or *H. zacatecana* (MEX-39, MEX-40, MEX-41) (50 nematodes/ml) on four second-instar *D. balteata* larvae previously fed on cucumber plants of the commercial varieties “T” (Tanja, dark grey), “S” (Sonja, light grey), “H” (Hokus, dark green) or “M76” (Marketmore 76, light green) for five days (n=15-20) . Bars indicate average (± SE). P values are given for treatments [generalized linear model (family, Binomial)] followed by pairwise comparisons of Least Squares Means (LSMeans). Not significant (n.s., p>0.05) and letters indicate significant differences between treatments (p<0.05)

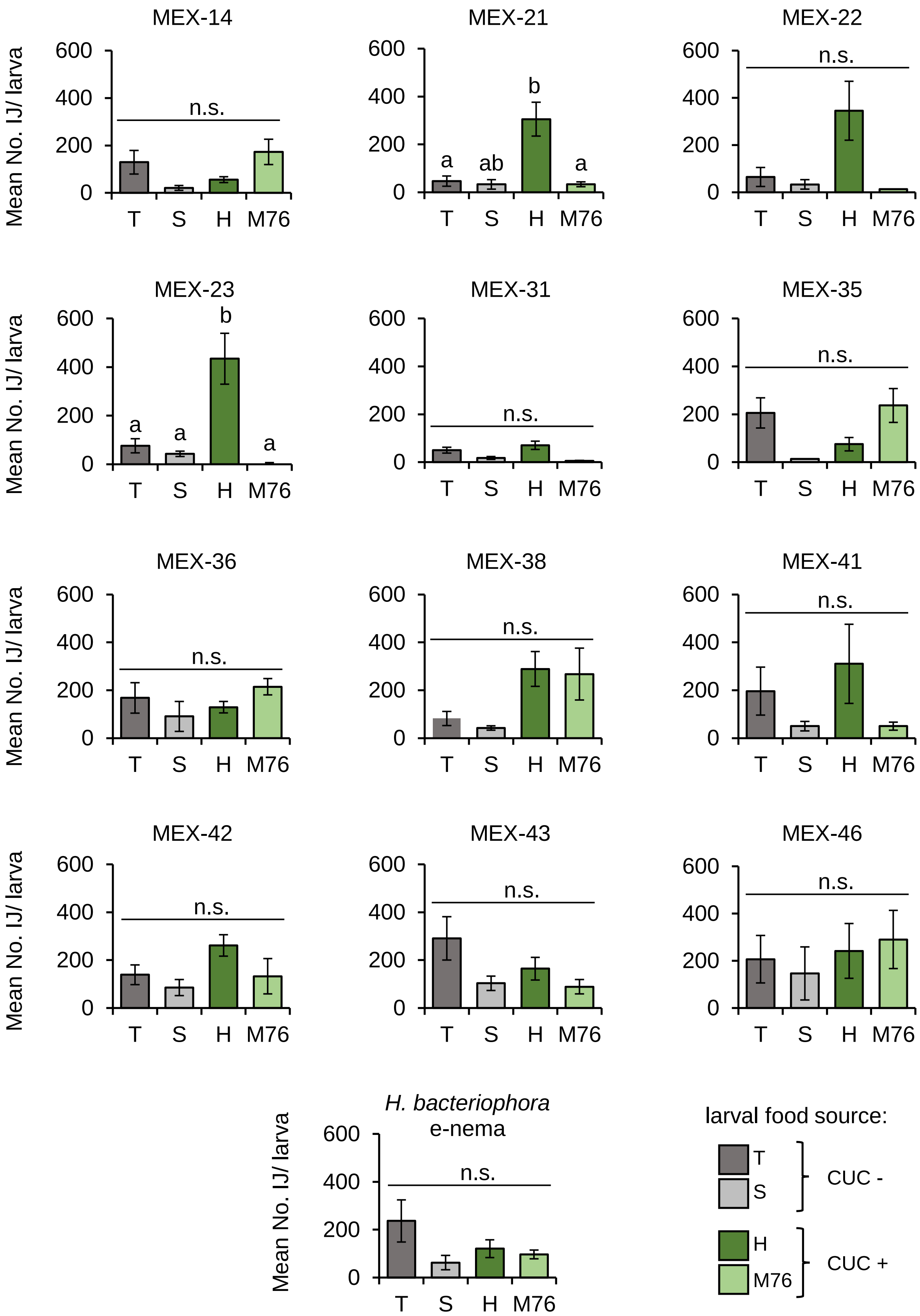

Fig. S5 Progeny of *Heterorhabditis* sp. entomopathogenic nematodes from *Diabrotica balteata* larvae fed on cucumber plants. Mean number (%) of Infective Juveniles (IJ) per *D. balteata* larva infected by 13 isolates of *Heterorhabditis* sp. entomopathogenic nematodes 15 days after inoculation with 500 ml of a suspension with *H. bacteriophora* or *H. zacatecana* (250 nematodes/ml) on four second-instar *D. balteata* larvae (n=2-10). Prior inoculation (n=10), the larvae fed for five days on cucumber plants of the commercial varieties "T" (Tanja, dark grey), "S" (Sonja, light grey), "H" (Hokus, dark green) or "M76" (Marketmore 76, light green). Bars indicate average (± SE). P values are given for treatments [generalized linear model (family, Binomial)] followed by pairwise comparisons of Least Squares Means (LSMeans). Not significant (n.s., $p>0.05$) and letters indicate significant differences between treatments ($p<0.05$)

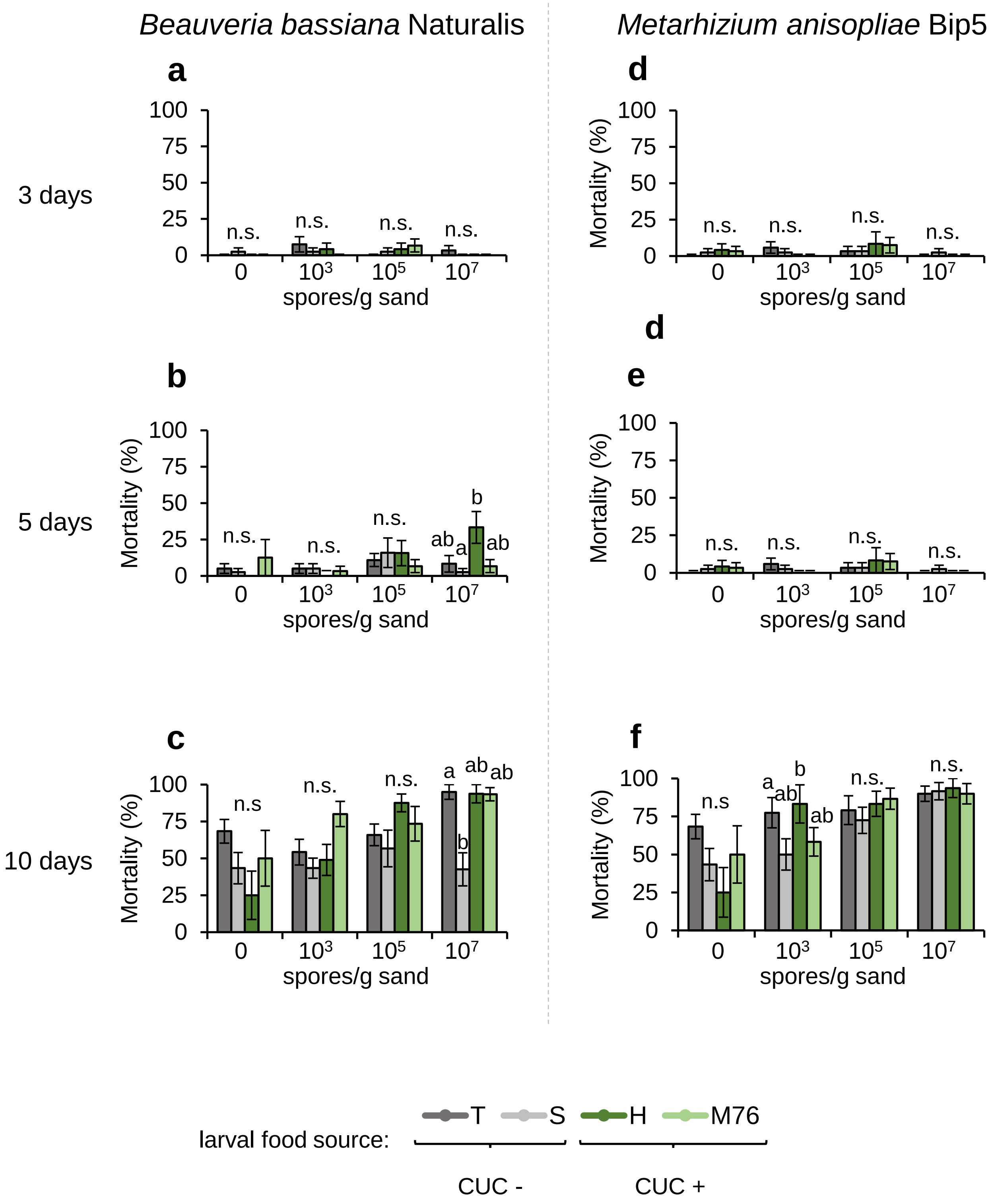

Fig. S6 Cucurbitacins do not protect *Diabrotica balteata* larvae against entomopathogenic fungi.
Second-instar *D. balteata* larvae fed for five days on cucumber plants of the commercial varieties "T" (Tanja, dark grey), "S" (Sonja, light grey), "H" (Hokus, dark green) or "M76" (Marketmore 76, light green) and then were exposed to entomopathogenic fungi. Mortality (%) of four *D. balteata* larvae infected with *B. bassiana* Naturalis (**a**-**c**) or *M. anisopliae* Bip5 (**d**-**f**, n=30) after three (**a**, **d**), five (**b**, **e**) or ten days (**c**, **f**) (n=30). Bars indicate average (± SE). *P* values are given for treatments [generalized linear model (family, Binomial)] followed by pairwise comparisons of Least Squares Means (LSMeans). Not significant (n.s., p>0.05). Different letters indicate significant differences among plant varieties, p<0.05

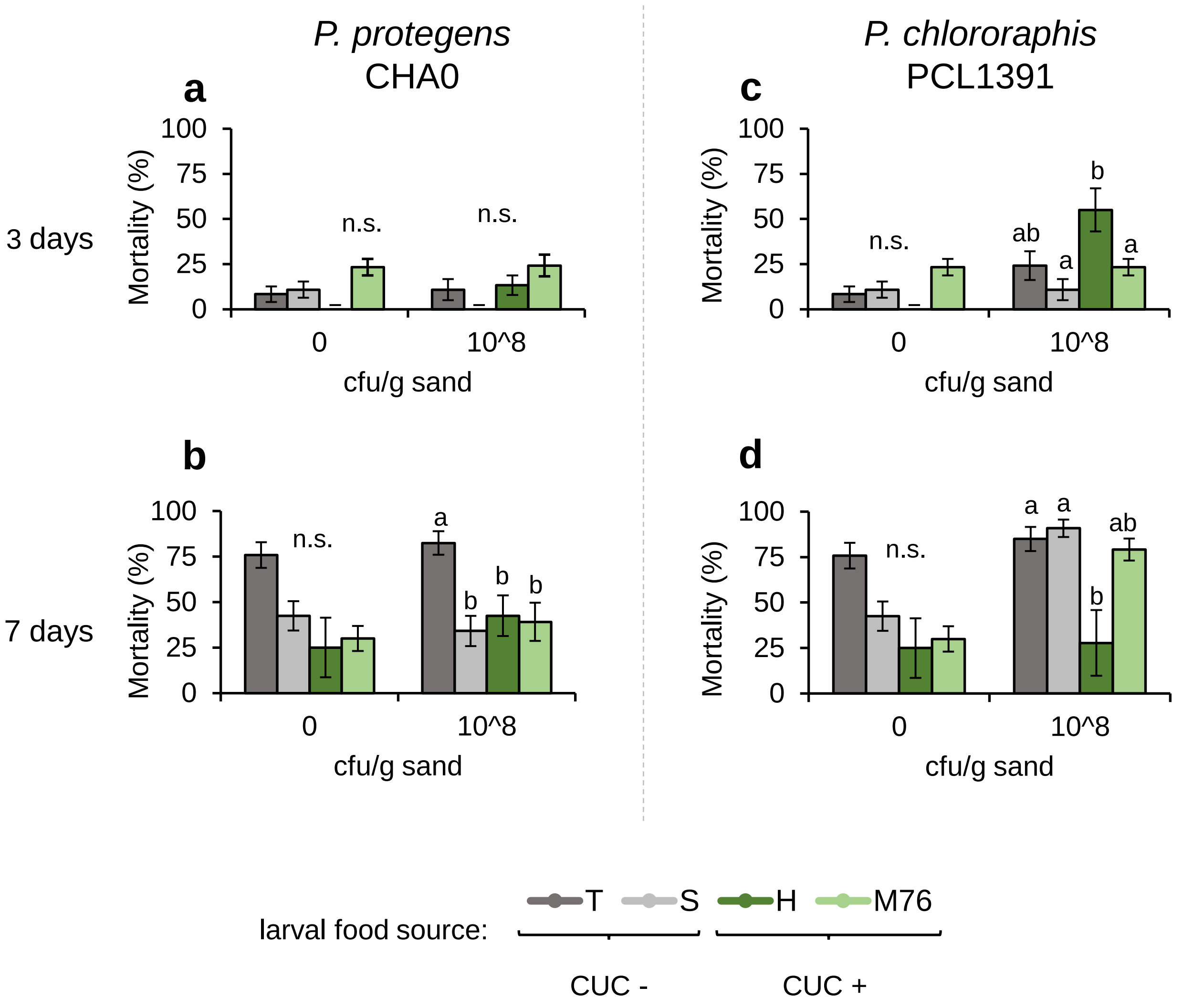

Fig. S7 Cucurbitacins do not protect *Diabrotica balteata* larvae against entomopathogenic bacteria.
Second-instar *D. balteata* larvae fed for five days on cucumber plants of the commercial varieties "T" (Tanja, dark grey), "S" (Sonja, light grey), "H" (Hokus, dark green) or "M76" (Marketmore 76, light green) and then were exposed to entomopathogenic bacteria. Mortality (%) of four *D. balteata* larvae infected with *P. protegens* CHA0 (**a**, **b**) or *P. chlororaphis* PCL1391 (**c**, **d**) after three (**a**, **c**) or seven days (**b**, **d**) (n=30). Bars indicate average (± SE). *P* values are given for treatments [generalized linear model (family, Binomial)] followed by pairwise comparisons of Least Squares Means (LSMeans). Not significant (n.s., p>0.05). Different letters indicate significant differences among plant varieties, p<0.05

Table S1 Putative identification of cucurbitacins found in plant and larval tissues. Cucurbitacins were putatively identified based on their retention times (RT), exact masses and relative mass defects (allowing for molecular formula determination, MF) and compared with those of the standard Cucurbitacin B as well as with available databases such as the Dictionary of Natural Product (CRC Press)

RT (min)	$(M+HCOO)^-$	$(M-H)^-$	Relative mass defect (RMD)	MF	Putative identification
2.52	769.4002	723.3948	546	$C_{38}H_{60}O_{13}$	Cucurbitacin IIa glucoside
2.44	767.3840	721.3792	526	$C_{38}H_{58}O_{13}$	Dihydrocucurbitacin B glucoside
2.16	725.3730	679.3730	549	$C_{36}H_{56}O_{12}$	Unknown Cucurbitacin #1
3.02	765.3694	719.3640	506	$C_{38}H_{56}O_{13}$	Cuc B glucoside
3.02	605.3328	559.3328	595	$C_{32}H_{48}O_8$	Cucurbitacin C
3.08	767.3834	721.3779	524	$C_{38}H_{58}O_{13}$	Isomer of dihydro-cucurbitacin B glucoside
2.44	727.3903	681.3848	565	$C_{36}H_{58}O_{12}$	Metabolized Cucurbitacin # 1
2.49	725.3757	679.3695	544	$C_{36}H_{56}O_{12}$	Deacetylated-dihydro-cucurbitacin B glucoside
2.52	723.3651	677.3601	532	$C_{36}H_{54}O_{12}$	Unknown Cucurbitacin # 2

Table S2 ANOVA results for the comparisons of cucurbitacin content in cucumber tissues of the variety T, undamaged or damaged by fifteen *D. balteata* larvae freely feeding on the plants for five days. Not significant (n.s., p>0.05)

Plant tissue	Putative cucurbitacin	Cucumber variety T								
		Undamaged			Damaged			F	p-value	symbol
		n	Mean (µg/g FW)	±SE	*n*	Mean (µg/g FW)	±SE			
roots	Cuc C	8	0.00	0.00	7	0.01	0.01	0.82	0.37	n.s.
	Unknown Cuc # 1	8	0.00	0.00	7	0.00	0.00	0.82	0.33	n.s.
	Unknown Cuc # 2	8	0.00	0.00	7	0.06	0.06	1.02	0.32	n.s.
	Deacetylated Dihydro Cuc B glu	8	0.00	0.00	7	0.00	0.00	14.00	0.00	n.s.
	Metabolized Cuc # 1	8	0.00	0.00	7	0.00	0.00	0.00	0.00	n.s.
	Cuc B glu	8	0.00	0.00	7	0.00	0.00	1.00	0.33	n.s.
	Dihydro Cuc B glu	8	0.10	0.03	7	0.14	0.11	1.30	0.33	n.s.
	Isomer of Dihydro Cuc B glu	8	0.00	0.00	7	0.00	0.00	1.00	0.33	n.s.
	Cuc II a glu	8	0.00	0.00	7	0.00	0.00	1.00	0.33	n.s.
	**TOTAL**	**8**	**0.10**	**0.03**	**7**	**0.22**	**0.19**	**1.81**	**0.19**	**n.s.**
stems	Cuc C	8	0.00	0.00	8	0.00	0.00	0.36	0.55	n.s.
	Unknown Cuc # 1	8	0.00	0.00	8	0.00	0.00	0.00	0.00	n.s.
	Unknown Cuc # 2	8	0.00	0.00	8	0.01	0.01	1.00	0.33	n.s.
	Deacetylated Dihydro Cuc B glu	8	0.00	0.00	8	0.00	0.00	0.00	0.00	n.s.
	Metabolized Cuc # 1	8	0.00	0.00	8	0.00	0.00	0.00	0.00	n.s.
	Cuc B glu	8	0.00	0.00	8	0.00	0.00	0.00	0.00	n.s.
	Dihydro Cuc B glu	8	0.36	0.12	8	0.24	0.11	0.56	0.46	n.s.
	Isomer of Dihydro Cuc B glu	8	0.00	0.00	8	0.00	0.00	0.00	0.00	n.s.
	Cuc II a glu	8	0.01	0.01	8	0.01	0.01	0.03	0.87	n.s.
	**TOTAL**	**8**	**0.37**	**0.13**	**8**	**0.26**	**0.13**	**0.43**	**0.51**	**n.s.**
cotyledons	Cuc C	8	0.00	0.00	8	0.00	0.00	1.00	0.33	n.s.
	Unknown Cuc # 1	8	0.00	0.00	8	0.00	0.00	0.00	0.00	n.s.
	Unknown Cuc # 2	8	0.01	0.01	8	0.00	0.00	1.00	0.33	n.s.
	Deacetylated Dihydro Cuc B glu	8	0.00	0.00	8	0.00	0.00	0.00	0.00	n.s.
	Metabolized Cuc # 1	8	0.00	0.00	8	0.00	0.00	0.00	0.00	n.s.
	Cuc B glu	8	0.00	0.00	8	0.00	0.00	0.00	0.00	n.s.
	Dihydro Cuc B glu	8	0.19	0.06	8	0.12	0.03	0.81	0.38	n.s.
	Isomer of Dihydro Cuc B glu	8	0.00	0.00	8	0.00	0.00	0.00	0.00	n.s.
	Cuc II a glu	8	0.00	0.00	8	0.00	0.00	0.00	0.00	n.s.
	**TOTAL**	**8**	**0.20**	**0.07**	**8**	**0.12**	**0.03**	**1.20**	**0.29**	**n.s.**
leaves	Cuc C	7	0.00	0.00	7	0.00	0.00	2.39	0.14	n.s.
	Unknown Cuc # 1	7	0.00	0.00	7	0.00	0.00	0.00	0.00	n.s.
	Unknown Cuc # 2	7	0.00	0.00	7	0.01	0.01	0.758	0.40	n.s.
	Deacetylated Dihydro Cuc B glu	7	0.00	0.00	7	0.00	0.00	1.00	0.33	n.s.
	Metabolized Cuc # 1	7	0.00	0.00	7	0.00	0.00	0.00	0.00	n.s.
	Cuc B glu	7	0.00	0.00	7	0.00	0.00	0.00	0.00	n.s.
	Dihydro Cuc B glu	7	0.14	0.04	7	0.15	0.07	0.01	0.89	n.s.
	Isomer of Dihydro Cuc B glu	7	0.02	0.02	7	0.00	0.00	1.00	0.33	n.s.
	Cuc II a glu	7	0.00	0.00	7	0.00	0.00	0.60	0.45	n.s.
	**TOTAL**	**7**	**0.16**	**0.06**	**7**	**0.17**	**0.08**	**0.002**	**0.95**	**n.s.**

Table S3 ANOVA results for the comparisons of cucurbitacin content in cucumber tissues of the variety S, undamaged or damaged by fifteen *D. balteata* larvae freely feeding on the plants for five days. Not significant (n.s., p>0.05) and stars indicate differences between treatments: *p<0.05

Plant tissue	Putative identification of the cucurbitacin	Cucumber variety S								
		Undamaged			Damaged			F	p-value	symbol
		n	Mean (µg/g FW)	±SE	*n*	Mean (µg/g FW)	±SE			
roots	Cuc C	8	0.00	0.00	8	0.00	0.00	1.00	0.33	n.s.
	Unknown Cuc # 1	8	0.00	0.00	8	0.00	0.00	1.00	0.33	n.s.
	Unknown Cuc # 2	8	0.00	0.00	8	0.01	0.01	1.00	0.33	n.s.
	Deacetylated Dihydro Cuc B glu	8	0.00	0.00	8	0.00	0.00	0.00	0.00	n.s.
	Metabolized Cuc # 1	8	0.00	0.00	8	0.00	0.00	0.00	0.00	n.s.
	Cuc B glu	8	0.00	0.00	8	0.00	0.00	1.00	0.33	n.s.
	Dihydro Cuc B glu	8	0.19	0.05	8	0.11	0.03	0.01	0.90	n.s.
	Isomer of Dihydro Cuc B glu	8	0.00	0.00	8	0.00	0.00	0.00	0.00	n.s.
	Cuc II a glu	8	0.01	0.01	8	0.00	0.00	0.24	0.24	n.s.
	**TOTAL**	**8**	**0.21**	**0.06**	**8**	**0.12**	**0.04**	**0.02**	**0.88**	**n.s.**
stems	Cuc C	7	0.00	0.00	9	0.00	0.00	1.15	0.30	n.s.
	Unknown Cuc # 1	7	0.00	0.00	9	0.00	0.00	0.00	0.00	n.s.
	Unknown Cuc # 2	7	0.00	0.00	9	0.00	0.00	0.00	0.00	n.s.
	Deacetylated Dihydro Cuc B glu	7	0.00	0.00	9	0.00	0.00	0.00	0.00	n.s.
	Metabolized Cuc # 1	7	0.00	0.00	9	0.00	0.00	0.00	0.00	n.s.
	Cuc B glu	7	0.00	0.00	9	0.00	0.00	0.00	0.00	n.s.
	Dihydro Cuc B glu	7	0.33	0.13	9	0.18	0.07	0.85	0.00	n.s.
	Isomer of Dihydro Cuc B glu	7	0.00	0.00	9	0.00	0.00	0.00	0.00	n.s.
	Cuc II a glu	7	0.00	0.00	9	0.01	0.01	0.55	0.55	n.s.
	**TOTAL**	**7**	**0.00**	**0.13**	**81**	**0.01**	**0.07**	**0.77**	**0.39**	**n.s.**
cotyledons	Cuc C	8	0.01	0.00	8	0.00	0.00	2.97	0.10	n.s.
	Unknown Cuc # 1	8	0.00	0.00	8	0.00	0.00	0.004	0.94	n.s.
	Unknown Cuc # 2	8	0.00	0.00	8	0.12	0.12	0.88	0.36	n.s.
	Deacetylated Dihydro Cuc B glu	8	0.00	0.00	8	0.00	0.00	1.13	0.30	n.s.
	Metabolized Cuc # 1	8	0.00	0.00	8	0.00	0.00	0.00	0.00	n.s.
	Cuc B glu	8	0.00	0.00	8	0.00	0.00	0.00	0.00	n.s.
	Dihydro Cuc B glu	8	0.37	0.08	8	0.16	0.04	7.30	0.01	*
	Isomer of Dihydro Cuc B glu	8	0.03	0.03	8	0.00	0.00	1.13	0.30	n.s.
	Cuc II a glu	8	0.01	0.01	8	0.00	0.00	2.31	0.14	n.s.
	**TOTAL**	**8**	**0.42**	**0.12**	**8**	**0.28**	**0.16**	**1.43**	**0.24**	**n.s.**
leaves	Cuc C	7	0.00	0.00	8	0.01	0.01	1.14	0.30	n.s.
	Unknown Cuc # 1	7	0.00	0.00	8	0.00	0.00	0.00	0.00	n.s.
	Unknown Cuc # 2	7	0.00	0.00	8	0.00	0.00	0.00	0.00	n.s.
	Deacetylated Dihydro Cuc B glu	7	0.00	0.00	8	0.00	0.00	0.00	0.00	n.s.
	Metabolized Cuc # 1	7	0.00	0.00	8	0.00	0.00	0.00	0.00	n.s.
	Cuc B glu	7	0.00	0.00	8	0.00	0.00	0.00	0.00	n.s.
	Dihydro Cuc B glu	7	0.24	0.09	8	0.25	0.09	0.001	0.97	n.s.
	Isomer of Dihydro Cuc B glu	7	0.00	0.00	8	0.00	0.00	0.86	0.36	n.s.
	Cuc II a glu	7	0.00	0.00	8	0.01	0.00	4.34	0.05	n.s.
	**TOTAL**	**7**	**0.24**	**0.10**	**8**	**0.27**	**0.10**	**0.02**	**0.87**	**n.s.**

Table S4 ANOVA results for the comparisons of cucurbitacin content in cucumber tissues of the variety H, undamaged or damaged by fifteen *D. balteata* larvae freely feeding on the plants for five days. Not significant (n.s., p>0.05) and stars indicate differences between treatments: *p<0.05

Plant tissue	Putative identification of the cucurbitacin	Cucumber variety H								
		Undamaged			Damaged			F	p-value	symbol
		n	Mean (µg/g FW)	±SE	*n*	Mean (µg/g FW)	±SE			
roots	Cuc C	8	0.00	0.00	8	0.01	0.00	0.86	0.36	n.s.
	Unknown Cuc # 1	8	0.01	0.00	8	0.01	0.00	0.00	0.95	n.s.
	Unknown Cuc # 2	8	0.00	0.00	8	0.00	0.00	0.88	0.36	n.s.
	Deacetylated Dihydro Cuc B glu	8	0.00	0.00	8	0.00	0.00	0.88	0.36	n.s.
	Metabolized Cuc # 1	8	0.00	0.00	8	0.00	0.00	0.00	0.00	n.s.
	Cuc B glu	8	0.13	0.06	8	0.18	0.05	0.39	0.53	n.s.
	Dihydro Cuc B glu	8	0.30	0.08	8	0.40	0.07	1.00	0.33	n.s.
	Isomer of Dihydro Cuc B glu	8	0.00	0.00	8	0.01	0.01	1.96	0.18	n.s.
	Cuc II a glu	8	0.02	0.01	8	0.04	0.01	2.87	0.11	n.s.
	**TOTAL**	**8**	**0.46**	**0.15**	**8**	**0.65**	**0.15**	**1.11**	**0.30**	**n.s.**
stems	Cuc C	8	0.04	0.01	9	0.31	0.11	5.21	0.037	*
	Unknown Cuc # 1	8	0.00	0.00	9	0.00	0.00	0.96	0.34	n.s.
	Unknown Cuc # 2	8	0.00	0.00	9	0.00	0.00	1.63	0.22	n.s.
	Deacetylated Dihydro Cuc B glu	8	0.01	0.00	9	0.01	0.00	0.25	0.61	n.s.
	Metabolized Cuc # 1	8	0.00	0.00	9	0.00	0.00	0.00	0.00	n.s.
	Cuc B glu	8	0.15	0.03	9	0.19	0.04	0.83	0.37	n.s.
	Dihydro Cuc B glu	8	5.02	0.50	9	4.26	0.43	1.32	0.26	n.s.
	Isomer of Dihydro Cuc B glu	8	0.07	0.02	9	0.06	0.02	0.17	0.68	n.s.
	Cuc II a glu	8	3.15	0.22	9	3.20	0.28	0.01	0.89	n.s.
	**TOTAL**	**8**	**8.44**	**0.78**	**9**	**8.04**	**0.88**	**0.14**	**0.71**	**n.s.**
cotyledons	Cuc C	7	0.72	0.29	7	2.87	1.15	3.30	0.09	n.s.
	Unknown Cuc # 1	7	0.00	0.00	7	0.00	0.00	0.00	0	n.s.
	Unknown Cuc # 2	7	0.04	0.02	7	0.02	0.02	0.97	0.34	n.s.
	Deacetylated Dihydro Cuc B glu	7	0.00	0.00	7	0.00	0.00	0.00	0.00	n.s.
	Metabolized Cuc # 1	7	0.00	0.00	7	0.00	0.00	0.00	0.00	n.s.
	Cuc B glu	7	0.00	0.00	7	0.00	0.00	0.00	0.00	n.s.
	Dihydro Cuc B glu	7	27.41	2.03	7	24.47	0.63	1.92	0.19	n.s.
	Isomer of Dihydro Cuc B glu	7	0.00	0.00	7	0.00	0.00	1.00	0.33	n.s.
	Cuc II a glu	7	7.13	0.63	7	6.73	0.37	0.30	0.59	n.s.
	**TOTAL**	**7**	**35.30**	**2.96**	**7**	**34.09**	**2.16**	**0.17**	**0.68**	**n.s.**
leaves	Cuc C	7	4.90	2.60	8	7.09	0.95	0.62	0.447	n.s.
	Unknown Cuc # 1	7	0.00	0.00	8	0.00	0.00	0.00	0.00	n.s.
	Unknown Cuc # 2	7	0.09	0.05	8	0.09	0.09	0.0006	0.98	n.s.
	Deacetylated Dihydro Cuc B glu	7	0.00	0.00	8	0.00	0.00	0.00	0.00	n.s.
	Metabolized Cuc # 1	7	0.00	0.00	8	0.00	0.00	0.00	0.00	n.s.
	Cuc B glu	7	0.00	0.00	8	0.00	0.00	0.00	0.00	n.s.
	Dihydro Cuc B glu	7	55.79	3.35	8	52.70	4.14	0.33	0.57	n.s.
	Isomer of Dihydro Cuc B glu	7	0.00	0.00	8	0.00	0.00	1.00	0.33	n.s.
	Cuc II a glu	7	4.62	0.80	8	5.29	0.63	0.05	0.81	n.s.
	**TOTAL**	**7**	**65.40**	**6.80**	**8**	**65.17**	**5.80**	**0.02**	**0.87**	**n.s.**

Table S5 ANOVA results for the comparisons of cucurbitacin content in cucumber tissues of the variety M76, undamaged or damaged by fifteen *D. balteata* larvae freely feeding on the plants for five days. Not significant (n.s., p>0.05) and stars indicate differences between treatments: *p<0.05, **p<0.01

**Plant tissue**	**Putative identification of the cucurbitacin**	**Cucumber variety M76**								
		Undamaged			Damaged					
		n	**Mean (µg/g FW)**	**±SE**	***n***	**Mean (µg/g FW)**	**±SE**	**F**	**p-value**	**symbol**
**roots**	Cuc C	8	0.00	0.00	8	0.00	0.00	0.05	0.81	n.s.
	Unknown Cuc # 1	8	0.01	0.00	8	0.02	0.01	1.29	0.27	n.s.
	Unknown Cuc # 2	8	0.00	0.00	8	0.00	0.00	0.00	0.00	n.s.
	Deacetylated Dihydro Cuc B glu	8	0.00	0.00	8	0.00	0.00	0.0005	0.99	n.s.
	Metabolized Cuc # 1	8	0.00	0.00	8	0.00	0.00	0.00	0.00	n.s.
	Cuc B glu	8	0.24	0.09	8	0.32	0.09	0.41	0.53	n.s.
	Dihydro Cuc B glu	8	0.44	0.15	8	0.34	0.15	0.22	0.64	n.s.
	Isomer of Dihydro Cuc B glu	8	0.03	0.03	8	0.03	0.02	0.01	0.89	n.s.
	Cuc II a glu	8	0.06	0.02	8	0.02	0.01	1.62	0.22	n.s.
	**TOTAL**	**8**	**0.78**	**0.29**	**8**	**0.74**	**0.28**	**0.01**	**0.90**	**n.s.**
**stems**	Cuc C	7	0.10	0.03	8	0.53	0.31	1.64	0.22	n.s.
	Unknown Cuc # 1	7	0.00	0.00	8	0.01	0.01	0.34	0.56	n.s.
	Unknown Cuc # 2	7	0.01	0.01	8	0.00	0.00	1.15	0.30	n.s.
	Deacetylated Dihydro Cuc B glu	7	0.00	0.00	8	0.00	0.00	0.56	0.46	n.s.
	Metabolized Cuc # 1	7	0.00	0.00	8	0.00	0.00	0.00	0.00	n.s.
	Cuc B glu	7	0.24	0.05	8	0.23	0.06	0.02	0.88	n.s.
	Dihydro Cuc B glu	7	5.95	0.57	8	5.81	0.77	0.02	0.88	n.s.
	Isomer of Dihydro Cuc B glu	7	0.06	0.01	8	0.03	0.01	4.06	0.06	n.s.
	Cuc II a glu	7	3.46	0.38	8	3.40	0.33	0.01	0.91	n.s.
	**TOTAL**	**7**	**9.82**	**1.06**	**8**	**10.00**	**1.49**	**0.01**	**0.9**	**n.s.**
**cotyledons**	Cuc C	8	0.98	0.36	8	3.09	1.36	2.25	0.15	n.s.
	Unknown Cuc # 1	8	0.00	0.00	8	0.00	0.00	1.00	0.33	n.s.
	Unknown Cuc # 2	8	0.07	0.03	8	0.20	0.11	1.32	0.26	n.s.
	Deacetylated Dihydro Cuc B glu	8	0.00	0.00	8	0.00	0.00	0.00	0.00	n.s.
	Metabolized Cuc # 1	8	0.00	0.00	8	0.00	0.00	0.00	0.00	n.s.
	Cuc B glu	8	0.00	0.00	8	0.00	0.00	0.12	0.72	n.s.
	Dihydro Cuc B glu	8	25.65	0.50	8	27.34	1.38	1.32	0.26	n.s.
	Isomer of Dihydro Cuc B glu	8	0.02	0.02	8	0.00	0.00	1.00	0.33	n.s.
	Cuc II a glu	8	5.28	0.32	8	6.48	0.40	5.60	0.03	*
	**TOTAL**	**8**	**31.99**	**1.22**	**8**	**37.11**	**3.25**	**3.40**	**0.08**	**n.s.**
**leaves**	Cuc C	8	2.09	1.00	8	6.21	1.42	5.66	0.03	*
	Unknown Cuc # 1	8	0.00	0.00	8	0.00	0.00	1	0.33	n.s.
	Unknown Cuc # 2	8	0.04	0.02	8	0.11	0.06	1.05	0.32	n.s.
	Deacetylated Dihydro Cuc B glu	8	0.00	0.00	8	0.00	0.00	0.00	0.00	n.s.
	Metabolized Cuc # 1	8	0.00	0.00	8	0.00	0.00	0.00	0.00	n.s.
	Cuc B glu	8	0.00	0.00	8	0.00	0.00	0.00	0.00	n.s.
	Dihydro Cuc B glu	8	40.24	4.70	8	49.43	4.02	2.20	0.15	n.s.
	Isomer of Dihydro Cuc B glu	8	0.00	0.00	8	0.00	0.00	0.00	0.00	n.s.
	Cuc II a glu	8	2.28	0.34	8	4.00	0.42	10.06	0.006	**
	**TOTAL**	**8**	**44.65**	**6.06**	**8**	**59.75**	**5.92**	**3.80**	**0.07**	**n.s.**

Table S6 ANOVA results for the comparisons of content of cucurbitacin sequestered by *D. balteata* larvae fed on cucumber full plants (shoots + roots), or only on roots or shoots for five days. P values are given for treatments [generalized linear model (family, Gaussian)] followed by pairwise comparisons of Least Squares Means (LSMeans). Letters indicate significant differences between treatments (p<0.05)

Putative identification of the cucurbitacin	Shoots + Roots				Roots				Shoots				F	p-value
	n	Mean (µg/g FW)	±SE	group	*n*	Mean (µg/g FW)	±SE	group	*n*	Mean (µg/g FW)	±SE	group		
**T-fed *D. balteata* larvae**														
Cuc C	5	0.01	0.01	a	5	0.00	0.00	a	5	0.00	0.00	a	2.64	0.11
Unknown Cuc # 1	5	0.00	0.00	a	5	0.00	0.00	a	5	0.00	0.00	a	1.00	0.39
Unknown Cuc # 2	5	0.00	0.00	a	5	0.00	0.00	a	5	0.00	0.00	a	0.00	0.00
Deacetylated Dihydro Cuc B glu	5	0.01	0.01	a	5	0.00	0.00	a	5	0.00	0.00	a	2.66	0.11
Metabolized Cuc # 1	5	0.00	0.00	a	5	0.00	0.00	a	5	0.00	0.00	a	1.00	0.39
Cuc B glu	5	0.02	0.00	a	5	0.00	0.00	b	5	0.00	0.00	b	15.75	0.001
Dihydro Cuc B glu	5	0.14	0.03	a	5	0.04	0.04	b	5	0.01	0.01	b	5.60	0.01
Isomer of Dihydro Cuc B glu	5	0.00	0.00	a	5	0.00	0.00	a	5	0.00	0.00	a	1.00	0.39
Cuc II a glu	5	0.04	0.01	a	5	0.00	0.00	b	5	0.00	0.00	b	12.38	0.001
**TOTAL**	5	0.23	0.07	a	5	0.04	0.04	b	5	0.01	0.01	b	10.76	0.002
**S-fed *D. balteata* larvae**														
Cuc C	5	0.02	0.01	a	5	0.00	0.00	b	5	0.00	0.00	b	5.72	0.01
Unknown Cuc # 1	5	0.01	0.01	a	5	0.00	0.00	a	5	0.00	0.00	a	1.00	0.39
Unknown Cuc # 2	5	0.01	0.01	a	5	0.00	0.00	a	5	0.00	0.00	a	2.39	0.13
Deacetylated Dihydro Cuc B glu	5	0.01	0.01	a	5	0.00	0.00	a	5	0.00	0.00	a	2.56	0.11
Metabolized Cuc # 1	5	0.00	0.00	a	5	0.00	0.00	a	5	0.00	0.00	a	0.00	0.00
Cuc B glu	5	0.01	0.01	a	5	0.00	0.00	a	5	0.00	0.00	a	2.65	0.11
Dihydro Cuc B glu	5	0.07	0.01	a	5	0.08	0.08	a	5	0.00	0.00	a	0.89	0.43
Isomer of Dihydro Cuc B glu	5	0.01	0.01	a	5	0.00	0.00	a	5	0.00	0.00	a	2.51	0.12
Cuc II a glu	5	0.03	0.00	a	5	0.00	0.00	b	5	0.00	0.00	b	83.23	<0.001
**TOTAL**	5	0.17	0.06	a	5	0.08	0.08	a	5	0.00	0.00	a	2.73	0.10
**H-fed *D. balteata* larvae**														
Cuc C	5	0.03	0.00	a	5	0.00	0.00	b	5	0.00	0.00	b	45.23	<0.001
Unknown Cuc # 1	5	5.45	2.48	a	5	2.48	0.27	ab	5	0.00	0.00	b	3.57	0.06
Unknown Cuc # 2	5	2.12	0.49	a	5	1.74	0.56	a	5	0.05	0.04	b	6.53	0.01
Deacetylated Dihydro Cuc B glu	5	16.26	4.02	a	5	20.08	3.26	a	5	1.44	0.40	b	10.76	0.002
Metabolized Cuc # 1	5	4.26	1.01	a	5	1.31	0.26	b	5	1.67	0.44	b	6.05	0.01
Cuc B glu	5	2.40	0.98	a	5	3.61	0.48	a	5	0.01	0.01	b	8.49	0.005
Dihydro Cuc B glu	5	0.07	0.02	a	5	0.00	0.00	b	5	0.00	0.00	b	11.48	0.002
Isomer of Dihydro Cuc B glu	5	3.59	1.09	a	5	2.13	0.31	ab	5	0.00	0.00	b	7.66	0.007
Cuc II a glu	5	0.02	0.01	a	5	0.00	0.00	a	5	0.00	0.00	a	1.68	0.22
**TOTAL**	5	34.20	10.10	a	5	31.34	5.15	a	5	3.18	0.89	b	8.15	0.006
**M76-fed *D. balteata* larvae**														
Cuc C	5	0.02	0.00	a	5	0.00	0.00	b	5	0.00	0.00	b	111.01	<0.001
Unknown Cuc # 1	5	4.73	0.85	a	5	2.22	0.23	b	5	0.16	0.06	c	20.09	0.00
Unknown Cuc # 2	5	1.93	0.31	a	5	1.64	0.25	a	5	0.00	0.00	b	20.78	0.00
Deacetylated Dihydro Cuc B glu	5	15.56	2.43	a	5	15.78	2.27	a	5	0.69	0.16	b	20.25	0.00
Metabolized Cuc # 1	5	4.99	1.21	a	5	1.17	0.44	b	5	1.48	0.15	b	8.00	0.006
Cuc B glu	5	3.32	1.46	a	5	2.42	0.73	a	5	0.27	0.11	a	2.75	0.10
Dihydro Cuc B glu	5	0.03	0.02	a	5	0.00	0.00	a	5	0.00	0.00	a	3.62	0.05
Isomer of Dihydro Cuc B glu	5	3.97	1.76	a	5	1.13	0.16	ab	5	0.04	0.02	b	3.96	0.04
Cuc II a glu	5	0.01	0.01	a	5	0.00	0.00	b	5	0.00	0.00	b	5.34	0.02
**TOTAL**	5	34.57	8.04	a	5	24.36	4.07	a	5	2.65	0.50	b	12.02	0.001

Table S7 ANOVA results for the comparisons of content of cucurbitacin sequestered by *D. balteata* after the larvae fed freely on full cucumber plants for 3, 5 and 7 days. P values are given for treatments [generalized linear model (family, Gaussian)] followed by pairwise comparisons of Least Squares Means (LSMeans). Letters indicate significant differences between treatments (p<0.05)

Time (days)	Putative cucurbitacin	T-fed *D. balteata* larvae				S-fed *D. balteata* larvae				H-fed *D. balteata* larvae				M76-fed *D. balteata* larvae				F	p-value
		n	Mean (µg/g FW)	±SE	group	*n*	Mean (µg/g FW)	±SE	group	*n*	Mean (µg/g FW)	±SE	group	*n*	Mean (µg/g FW)	±SE	group		
**3**	Cuc C	9	0.00	0.00	a	8	0.06	0.03	a	8	0.02	0.02	a	9	0.03	0.02	a	1.62	0.21
	Unknown Cuc # 1	9	0.00	0.00	a	8	0.04	0.02	a	8	0.33	0.16	a	9	0.74	0.43	a	2.05	0.13
	Unknown Cuc # 2	9	0.02	0.02	a	8	0.02	0.02	a	8	0.45	0.15	ab	9	0.85	0.36	b	3.84	0.02
	Deacetylated Dihydro Cuc B glu	9	0.04	0.02	a	8	0.05	0.03	a	8	7.50	1.93	b	9	12.25	2.03	b	18.66	<0.001
	Metabolized Cuc # 1	9	0.02	0.02	a	8	0.04	0.03	a	8	0.72	0.23	ab	9	1.14	0.49	b	3.92	0.02
	Cuc B glu	9	0.01	0.01	a	8	0.04	0.02	a	8	0.15	0.09	a	9	0.25	0.10	a	2.56	0.07
	Dihydro Cuc B glu	9	0.02	0.02	a	8	0.02	0.02	a	8	0.03	0.02	a	9	0.03	0.02	a	0.20	0.90
	Isomer of Dihydro Cuc B glu	9	0.02	0.02	a	8	0.10	0.03	a	8	0.33	0.17	a	9	0.53	0.24	a	2.43	0.09
	Cuc II a glu	9	0.02	0.02	a	8	0.08	0.03	a	8	0.02	0.02	a	9	0.08	0.03	a	2.73	0.06
	**TOTAL**	**9**	**0.13**	**0.12**	**a**	**8**	**0.44**	**0.23**	**a**	**8**	**9.55**	**2.79**	**b**	**9**	**15.91**	**3.73**	**b**	**18.57**	**<0.001**
**5**	Cuc C	8	0.00	0.00	a	10	0.03	0.02	a	8	0.06	0.03	a	8	0.00	0.00	a	2.37	0.09
	Cuc C	8	0.00	0.00	a	10	0.03	0.02	a	8	1.70	0.56	b	8	0.60	0.25	a	7.54	<0.001
	Unknown Cuc # 1	8	0.04	0.02	a	10	0.09	0.02	a	8	0.78	0.21	b	8	0.84	0.27	b	7.17	<0.001
	Unknown Cuc # 2	8	0.08	0.03	a	10	0.08	0.03	a	8	10.56	2.34	b	8	13.95	1.03	b	35.43	<0.001
	Deacetylated Dihydro Cuc B glu	8	0.04	0.02	a	10	0.04	0.02	a	8	1.77	0.56	ab	8	1.95	0.69	b	6.43	0.002
	Metabolized Cuc # 1	8	0.00	0.00	a	10	0.02	0.02	a	8	0.62	0.39	a	8	0.07	0.07	a	2.47	0.08
	Cuc B glu	8	0.08	0.03	a	10	0.03	0.02	a	8	0.08	0.03	a	8	0.00	0.00	a	2.70	0.06
	Dihydro Cuc B glu	8	0.06	0.03	ab	10	0.02	0.02	a	8	0.45	0.16	ab	8	0.55	0.23	b	4.15	0.01
	Isomer of Dihydro Cuc B glu	8	0.02	0.02	a	10	0.04	0.02	a	8	0.05	0.03	a	8	0.00	0.00	a	1.42	0.26
	**TOTAL**	**8**	**0.30**	**0.15**	**a**	**10**	**0.37**	**0.18**	**a**	**8**	**16.07**	**4.30**	**b**	**8**	**17.96**	**2.53**	**b**	**55.21**	**<0.001**
**7**	Cuc C	5	0.00	0.00	a	5	0.03	0.03	a	5	0.00	0.00	a	5	0.00	0.00	a	0.02	0.41
	Cuc C	5	0.03	0.03	a	5	0.00	0.00	a	5	6.75	1.41	b	5	7.19	2.96	b	6.01	0.01
	Unknown Cuc # 1	5	0.03	0.03	a	5	0.00	0.00	a	5	0.89	0.43	ab	5	1.39	0.58	b	3.55	0.04
	Unknown Cuc # 2	5	0.00	0.00	a	5	0.04	0.04	a	5	12.76	3.38	b	5	19.36	1.16	c	29.15	<0.001
	Deacetylated Dihydro Cuc B glu	5	0.00	0.00	a	5	0.06	0.04	a	5	1.52	0.76	a	5	1.62	0.77	a	2.73	0.08
	Metabolized Cuc # 1	5	0.00	0.00	a	5	0.07	0.04	a	5	5.20	1.92	a	5	4.45	2.38	a	3.31	0.05
	Cuc B glu	5	0.00	0.00	a	5	0.00	0.00	a	5	0.00	0.00	a	5	0.00	0.00	a	16.00	0.00
	Dihydro Cuc B glu	5	0.03	0.03	a	5	0.04	0.04	a	5	2.20	0.88	b	5	2.18	0.72	b	4.80	0.01
	Isomer of Dihydro Cuc B glu	5	0.00	0.00	a	5	0.00	0.00	a	5	0.00	0.00	a	5	0.00	0.00	a	16.00	0.00
	**TOTAL**	**5**	**0.08**	**0.08**	**a**	**5**	**0.23**	**0.18**	**a**	**5**	**29.33**	**8.77**	**b**	**5**	**36.19**	**8.58**	**b**	**18.20**	**<0.001**

Table S8 ANOVA results for the comparisons of weight gain by *D. balteata* larvae fed on cucumber full plants (shoots + roots), or only on roots or shoots for five days. P values are given for treatments [generalized linear model (family, Gaussian)] followed by pairwise comparisons of Least Squares Means (LSMeans). Letters indicate significant differences between treatments (p<0.05)

**Treatment**		**T-fed *D. balteata* larvae**				**S-fed *D. balteata* larvae**				**H-fed *D. balteata* larvae**				**M76-fed *D. balteata* larvae**				**F**	**p-value**
**Plant part consumed**	**Time (days)**	*n*	(mg/ larva)	±SE	group	*n*	(mg/ larva)	±SE	group	*n*	(mg/ larva)	±SE	group	*n*	(mg/ larva)	±SE	group		
Full plants	3	5	1.14	0.09	ab	5	1.27	0.06	b	5	0.97	0.09	a	5	0.90	0.03	a	5.35	0.009
	5	5	2.15	0.21	a	5	2.43	0.12	a	5	2.44	0.13	a	5	1.78	0.25	a	2.75	0.07
	total	5	3.28	0.30	ab	5	3.70	0.18	b	5	3.41	0.22	ab	5	2.68	0.28	a	4.32	0.02
Roots	3	5	0.95	0.11	a	5	1.07	0.10	a	5	0.45	0.02	b	5	0.53	0.06	b	13.90	<0.001
	5	5	0.79	0.15	a	5	0.52	0.14	a	5	0.89	0.04	a	5	0.81	0.07	a	1.97	0.15
	total	5	1.75	0.26	a	5	1.60	0.25	ab	5	1.34	0.06	b	5	1.34	0.13	b	4.91	0.01
Shoots	3	5	0.98	0.16	ab	5	0.69	0.09	a	5	1.19	0.09	b	5	0.94	0.07	ab	3.65	0.03
	5	5	0.43	0.08	a	5	0.75	0.13	a	5	0.45	0.11	a	5	0.69	0.05	a	2.93	0.06
	total	5	1.41	0.24	a	5	1.44	0.21	a	5	1.64	0.20	a	5	1.63	0.12	a	1.63	0.22

Table S9 ANOVA results for the comparisons of mortality rates of *D. balteata* larvae fed on cucumber full plants (shoots + roots), or only on roots or shoots for five days. P values are given for treatments [generalized linear model (family, binomial)] followed by pairwise comparisons of Least Squares Means (LSMeans). Letters indicate significant differences between treatments ($p<0.05$)

Treatment		T-fed *D. balteata* larvae				S-fed *D. balteata* larvae				H-fed *D. balteata* larvae				M76-fed *D. balteata* larvae				Chisq	p-value
**Plant part consumed**	**Time (days)**	*n*	Mortality (%)	±SE	group	*n*	Mortality (%)	±SE	group	*n*	Mortality (%)	±SE	group	*n*	Mortality (%)	±SE	group		
Full plants	3	5	0.00	0.00	a	5	2.67	1.63	a	5	1.33	1.33	a	5	0.00	0.00	a	2.56 E-10	1.00
	5	5	5.33	2.49	a	5	6.67	2.11	a	5	3.92	1.60	a	5	2.67	1.63	a	7.02	0.20
	total	5	5.33	2.49	a	5	9.33	3.74	a	5	5.25	2.93	a	5	2.67	1.63	a	17.65	0.23
Roots	3	5	5.33	2.49	a	5	4.00	1.63	a	5	6.67	3.65	a	5	3.92	1.60	a	2.56 E-10	1.00
	5	5	6.67	2.11	a	5	2.67	1.63	a	5	10.67	3.40	a	5	9.33	2.67	a	18.03	0.85
	total	5	12.00	4.60	a	5	6.67	3.27	a	5	17.33	7.05	a	5	13.25	4.27	a	15.70	0.201
Shoots	3	5	2.67	2.67	a	5	1.25	1.25	a	5	2.58	1.58	a	5	1.33	1.33	a	2.56 E-10	1.00
	5	5	5.33	2.49	a	5	3.92	1.60	a	5	1.33	1.33	a	5	6.67	3.65	a	16.82	0.86
	total	5	8.00	5.16	a	5	5.17	2.85	a	5	3.92	2.92	a	5	8.00	4.98	a	18.40	0.35

Table S10 ANOVA results for the comparisons of predation rates of *D. balteata* larvae 48 hours after exposure to insect predators. Previous exposure to predators, the larvae fed freely on cucumber plants of the varieties T, S, H or M76 for five days. P values are given for treatments [generalized linear model (family, binomial or quasibinomial)] followed by pairwise comparisons of Least Squares Means (LSMeans). Letters indicate significant differences between treatments (p<0.05)

Natural enemies	T-fed *D. balteata* larvae			S-fed *D. balteata* larvae			H-fed *D. balteata* larvae			M76-fed *D. balteata* larvae			Test	p-value
	n	Predation (%)	group	*n*	Predation (%)	group	*n*	Predation (%)	group	*n*	Predation (%)	group		
Dalotia coriaria	30	50	a	30	43.33	a	30	43.33	a	30	46.67	a	F=0.12	0.948
Chrysoperla carnea	30	76.67	a	30	76.67	a	30	83.33	a	30	83.33	a	Chisq= 119.26	0.841
Orius laevigatus	30	46.66	a	30	50	a	30	40	a	30	50	a	F=0.26	0.854

Table S11 ANOVA results for the comparisons of infection rates of *D. balteata* larvae five days after inoculation with 25 isolates of *Heterorhabditis* sp. entomopathogenic nematodes (EPN). Previous exposure to EPN, the larvae fed freely on cucumber plants of the varieties T, S, H or M76 for five days. P values are given for treatments [generalized linear model (family, binomial or quasibinomial)] followed by pairwise comparisons of Least Squares Means (LSMeans). Letters indicate significant differences between treatments (p<0.05)

Species	EPN isolate	T-fed *D. balteata* larvae				S-fed *D. balteata* larvae				H-fed *D. balteata* larvae				M76-fed *D. balteata* larvae				Test	p-value
		n	Infection (%)	±SE	group	*n*	Infection (%)	±SE	group	*n*	Infection (%)	±SE	group	*n*	Infection (%)	±SE	group		
Heterorhabditis bacteriophora	**MEX-14**	20	71.67	5.65	a	20	68.75	5.98	a	20	76.00	5.24	a	20	71.25	5.81	a	F=0.31	0.81
	**MEX-16**	15	62.22	5.80	a	17	59.41	5.62	a	20	65.00	5.26	a	20	71.75	4.45	a	Chisq= 74.02	0.46
	**MEX-17**	15	61.67	6.39	a	15	61.67	8.56	a	15	69.44	5.90	a	14	70.24	5.78	a	F=0.39	0.75
	**MEX-21**	20	39.58	6.31	a	20	41.67	7.05	a	20	42.50	5.89	a	20	62.92	6.55	a	F=2.84	0.04
	**MEX-22**	15	76.11	5.20	a	18	65.28	5.36	a	17	80.39	6.66	ab	20	97.08	2.03	b	Chisq= 66.97	<0.001
	**MEX-23**	15	51.67	8.26	a	17	71.86	4.26	ab	20	63.75	5.87	ab	20	74.50	4.62	b	F=2.78	0.04
	**MEX-29**	20	65.83	5.20	a	20	62.08	4.93	a	20	75.00	5.13	a	20	75.00	5.13	a	F=1.66	0.18
	**MEX-30**	15	55.00	8.86	a	17	54.41	6.15	a	20	67.08	6.55	a	20	55.50	6.00	a	F=0.78	0.51
	**MEX-31**	20	65.83	6.07	a	20	57.92	5.94	a	20	75.00	5.44	a	20	76.25	5.58	a	F=2.02	0.12
	**MEX-32**	15	52.78	9.95	a	15	44.44	9.47	a	15	57.78	9.18	a	15	53.33	8.90	a	F=0.23	0.87
	**MEX-33**	20	80.00	4.66	a	20	67.50	7.93	a	20	76.25	5.58	a	20	71.25	5.81	a	F=0.77	0.51
	**MEX-34**	15	65.56	9.23	a	18	75.00	6.84	a	17	87.75	6.19	a	20	89.58	3.91	a	F=2.55	0.06
	**MEX-35**	15	55.56	6.84	a	17	73.82	5.87	ab	20	80.50	4.13	b	20	59.00	5.86	a	F=3.94	0.01
	**MEX-36**	20	30.00	5.45	a	20	31.67	6.24	a	20	35.83	6.40	a	20	42.50	7.50	a	F=0.99	0.40
	**MEX-37**	20	65.00	6.64	a	20	72.50	5.10	a	20	69.58	5.00	a	19	66.23	5.96	a	F=0.33	0.79
	**MEX-38**	15	76.67	6.20	a	18	83.80	4.49	a	17	89.22	4.67	a	20	81.67	5.38	a	F=1.13	0.34
Heterorhabditis zacatecana	**MEX-39**	15	53.33	7.44	a	15	51.67	9.80	a	15	62.22	7.84	a	15	55.00	8.40	a	F=0.56	0.64
	**MEX-40**	15	63.89	5.37	a	14	57.74	8.89	a	14	73.81	5.92	a	15	68.89	7.78	a	F=1.29	0.28
	**MEX-41**	20	22.08	6.14	a	20	22.50	6.51	a	19	32.02	7.60	a	20	47.08	7.39	a	F=3.02	0.03
Heterorhabditis bacteriophora	**MEX-42**	20	39.58	6.31	a	18	78.24	5.52	b	17	77.94	5.80	b	20	90.83	3.87	b	F=14.15	<0.001
	**MEX-43**	15	61.11	5.85	a	18	81.94	4.87	a	16	65.10	7.50	a	15	78.33	6.84	a	F=2.53	0.06
	**MEX-44**	20	30.00	5.45	a	18	74.54	7.02	b	17	85.78	5.04	b	20	85.00	6.50	b	F=15.14	<0.001
	**MEX-45**	15	52.22	8.59	a	18	50.46	7.75	a	16	67.19	7.11	a	15	71.11	5.91	a	F=1.98	0.12
	**MEX-46**	20	26.67	6.00	a	20	42.50	7.60	ab	20	57.50	7.15	b	20	40.42	6.85	ab	F=4.04	0.01
	**eNema EF**	15	71.11	5.39	a	18	71.76	6.03	a	16	61.67	6.95	a	15	63.33	8.11	a	F=0.77	0.51
	**TOTAL**	**25**	**54.90**	**6.24**	**a**	**25**	**60.72**	**6.29**	**a**	**25**	**67.46**	**5.88**	**a**	**25**	**68.95**	**5.82**	**a**	**Chisq= 37.94**	**0.242**

Table S12 ANOVA results for the comparisons of mortality rates of *D. balteata* larvae 3, 5 and 7 days after inoculation with entomopathogenic fungi (EPF). Previous inoculation, the larvae fed freely on cucumber plants of the varieties T, S, H or M76 for five days. P values are given for treatments [generalized linear model (family, binomial or quasibinomial)] followed by pairwise comparisons of Least Squares Means (LSMeans). Letters indicate significant differences between treatments (p<0.05)

EPF Treatment	Conc.	Time (days)	T-fed *D. balteata* larvae				S-fed *D. balteata* larvae				H-fed *D. balteata* larvae				M76-fed *D. balteata* larvae				Test	p-value
			n	Mortality (%)	±SE	group	*n*	Mortality (%)	±SE	group	*n*	Mortality (%)	±SE	group	*n*	Mortality (%)	±SE	group		
**Negative control**	**0**	**3**	10	0.00	0.00	a	10	2.50	2.50	a	7	0.00	0.00	a	7	0.00	0.00	a	Chisq= 4.46	0.58
		**5**	10	5.00	3.33	a	10	2.50	2.50	a	7	0.00	0.00	a	8	12.50	12.50	a	Chisq= 17.59	0.59
		**7**	10	15.00	7.10	a	10	20.00	7.67	a	8	0.00	0.00	a	6	0	0.00	a	Chisq= 23.23	0.16
		**10**	10	68.33	8.03	a	10	43.33	10.59	a	8	25.00	16.36	a	8	50	18.89	a	Chisq= 42.45	0.11
***Beauveria bassiana* Naturalis**	10^3	**3**	10	7.50	5.33	a	10	2.50	2.50	a	8	4.166	4.16	a	10	0	0.00	a	Chisq= 20.52	0.35
		**5**	10	5.00	3.33	a	10	5.00	3.33	a	8	0.00	0.00	a	10	3.33	3.33	a	Chisq= 18.42	0.57
		**7**	10	5.83	3.93	a	10	5.83	3.93	a	8	14.58	7.34	a	10	33.33	12.94	a	F=2.95	0.04
		**10**	10	54.16	8.71	a	10	43.33	6.78	a	8	48.95	10.49	a	10	80.00	8.53	a	Chisq= 34.67	0.06
	10^5	**3**	10	0.00	0.00	a	10	2.50	2.50	a	8	4.16	4.16	a	10	6.66	4.44	a	Chisq= 15.58	0.27
		**5**	10	10.83	4.48	a	10	15.83	10.12	a	8	15.62	8.68	a	10	6.66	4.44	a	Chsiq= 38.50	0.79
		**7**	10	15.83	5.75	a	10	20.00	11.66	a	8	23.95	7.62	a	10	21.66	8.97	a	F=0.16	0.91
		**10**	10	65.83	7.29	a	10	56.66	12.41	a	8	87.50	6.09	a	10	73.33	11.70	a	F=1.90	0.14
	10^7	**3**	10	3.33	3.33	a	10	0.00	0.00	a	8	0.00	0.00	a	10	0	0.00	a	Chisq= 5.08	0.44
		**5**	10	8.33	5.69	ab	10	2.50	2.50	a	8	33.33	10.91	b	10	6.66	4.44	ab	Chisq= 34.53	0.008
		**7**	10	38.33	8.97	a	10	14.16	6.09	a	8	37.50	8.76	a	10	26.6	9.02	a	Chisq= 35.06	0.05
		10	10	95.00	5.00	a	10	42.50	11.14	b	8	93.75	6.25	ab	10	93.33	4.44	ab	Chisq= 29.05	<0.001
***Metarhizium anisopliae* BIPESCO5**	10^3	**3**	10	0.00	0.00	a	10	2.50	2.50	a	8	4.16	4.16	a	10	3.33	3.33	a	Chisq= 13.87	0.50
		**5**	10	5.00	3.33	a	10	11.66	6.93	a	8	4.16	4.16	a	10	3.33	3.33	a	Chisq= 28.32	0.64
		**7**	10	16.66	7.75	a	10	14.16	6.92	a	8	28.12	11.67	a	10	31.66	7.22	a	Chisq= 39.97	0.4885
		**10**	10	77.50	9.94	ab	10	50.00	10.24	a	8	83.33	12.59	b	10	58.33	9.37	ab	Chisq= 41.33	0.01
	10^5	**3**	10	5.80	3.93	a	10	2.50	2.50	a	8	0.00	0.00	a	10	0.00	0.00	a	Chisq= 11.64	0.29
		**5**	10	15.00	7.11	a	10	7.50	3.81	a	8	7.29	4.83	a	10	20.00	5.44	a	Chisq= 31.64	0.45
		**7**	10	30.00	8.88	a	10	26.66	9.92	a	8	25.00	7.71	a	10	36.66	11.05	a	F=0.29	0.82
		**10**	10	79.16	9.39	a	10	72.50	8.61	a	8	83.33	8.33	a	10	86.66	6.93	a	F=0.73	0.53
	10^7	**3**	10	3.33	3.33	a	10	3.33	3.33	a	8	8.33	8.33	a	10	7.5	5.33	a	Chisq= 27.21	0.61
		**5**	10	17.50	7.50	a	10	15.00	5.09	a	8	31.25	9.67	a	10	35	10.07	a	Chisq= 38.09	0.26
		**7**	10	60.00	9.60	a	10	53.33	8.16	a	8	37.50	12.89	a	10	36.66	11.60	a	F=1.2	0.31
		**10**	10	90.00	5.09	a	10	91.66	5.69	a	8	93.75	6.25	a	10	90	6.66	a	Chisq= 28.14	0.97

Table S13 ANOVA results for the comparisons of mortality rates of *D. balteata* larvae 3, 5 and 7 days after ingestion of cucumber germinates imbibed with entomopathogenic bacteria (EPP). Previous exposure, the larvae fed freely on cucumber plants of the varieties T, S, H or M76 for five days. P values are given for treatments [generalized linear model (family, binomial or quasibinomial)] followed by pairwise comparisons of Least Squares Means (LSMeans). Letters indicate significant differences between treatments (p<0.05)

EPP Treatment	Time (days)	T-fed *D. balteata* larvae				S-fed *D. balteata* larvae				H-fed *D. balteata* larvae				M76-fed *D. balteata* larvae				Test	p-value
		n	Mortality (%)	±SE	group	*n*	Mortality (%)	±SE	group	*n*	Mortality (%)	±SE	group	*n*	Mortality (%)	±SE	group		
CHA0	3	10	10.83	5.83	a	10	0.00	0.00	a	10	13.33	5.44	a	10	24.17	6.02	a	Chisq= 27.44	0.01
	5	10	29.17	5.86	a	10	25.00	7.14	a	10	26.67	8.31	a	10	25.00	6.09	a	Chisq= 34.59	0.95
	7	10	82.50	6.51	a	10	34.17	8.28	b	10	42.50	11.14	b	10	39.17	10.54	b	F=6.76	<0.001
PCL	3	10	24.17	8.00	ab	10	10.83	5.83	a	10	55.00	11.93	b	10	23.33	4.61	a	F=5.02	0.01
	5	10	40.00	10.60	a	10	46.67	11.86	a	6	41.67	14.77	a	10	45.83	10.19	a	F=0.11	0.95
	7	10	85.00	6.67	a	10	90.83	4.72	a	8	27.78	18.09	b	10	79.17	5.99	ab	Chisq= 33.95	0.02
Neg. control (water)	3	10	8.33	4.30	a	10	10.83	4.49	a	8	0.00	0.00	a	10	23.33	4.61	a	Chisq= 20.46	0.11
	5	10	12.50	6.72	a	10	32.50	8.47	a	8	12.50	12.50	a	10	12.50	5.59	a	Chsiq= 37.99	0.16
	7	10	75.83	7.08	a	10	42.50	8.09	a	8	25.00	16.37	a	10	30.00	6.94	a	Chisq= 34.71	0.00

Table S14 ANOVA results for the comparisons of EPN progeny of infected *D. balteata* larvae 15 days after inoculation with 13 isolates of *Heterorhabditis* sp. entomopathogenic nematodes (EPN). The progeny is expressed as average number of infective juveniles (Ijs) per infected *D. balteata* larva**.** Previous EPN inoculation, the larvae fed freely on cucumber plants of the varieties T, S, H or M76 for five days. P values are given for treatments [generalized linear model (family, binomial or quasibinomial)] followed by pairwise comparisons of Least Squares Means (LSMeans). Letters indicate significant differences between treatments (p<0.05)

**Treat.**	**T-fed *D. balteata* larvae**				**S-fed *D. balteata* larvae**				**H-fed *D. balteata* larvae**				**M76-fed *D. balteata* larvae**					
**EPN isolate**	*n*	Average IJs/ infected larva	±SE	group	*n*	Average IJs/ infected larva	±SE	group	*n*	Average IJs/larva	±SE	group	*n*	Average IJs/ infected larva	±SE	group	**F**	**p-value**
**MEX-14**	8	129.31	49.96	a	4	20.83	10.03	a	10	55.89	12.00	a	7	173.33	53.37	a	2.71	0.07
**MEX-21**	7	46.83	21.55	a	2	33.33	20.00	ab	10	305.44	70.57	b	6	33.33	10.18	a	6.30	0.00
**MEX-22**	7	65.24	40.47	a	6	74.44	28.31	a	10	345.44	124.96	a	5	13.33	0.00	a	2.98	0.05
**MEX-23**	10	76.22	29.10	a	7	42.38	10.95	a	10	434.44	104.81	b	2	20.00	6.67	a	7.30	0.00
**MEX-31**	8	50.14	12.11	a	5	17.33	6.22	a	10	70.33	17.23	a	3	5.56	1.70	a	3.09	0.05
**MEX-35**	8	205.83	62.79	a	3	13.33	0.00	a	5	75.11	28.41	a	10	236.89	70.81	a	1.85	0.17
**MEX-36**	10	168.00	63.10	a	3	91.11	62.58	a	10	129.11	23.97	a	10	214.67	33.76	a	0.97	0.42
**MEX-38**	8	81.81	29.58	a	6	42.22	9.38	a	9	288.89	72.48	a	8	267.50	108.04	a	2.98	0.05
**MEX-41**	7	196.19	100.07	a	6	50.37	19.82	a	9	310.37	164.98	a	9	50.37	16.78	a	1.47	0.25
**MEX-42**	10	138.89	41.20	a	8	85.28	33.29	a	10	261.00	44.73	a	7	132.70	73.30	a	2.60	0.07
**MEX-43**	9	290.86	90.98	a	10	103.22	30.00	a	10	164.67	47.34	a	8	88.61	29.91	a	2.69	0.06
**MEX-46**	8	206.67	100.29	a	6	146.67	112.24	a	10	241.89	116.40	a	9	290.37	123.74	a	0.24	0.87
**eNema EF**	4	236.67	87.67	a	8	62.50	30.31	a	10	120.67	37.08	a	10	96.89	18.36	a	2.66	0.07
**TOTAL**	**13**	**145.59**	**56.07**	**a**	**13**	**60.23**	**28.70**	**a**	**13**	**215.64**	**66.53**	**b**	**13**	**124.89**	**42.05**	**a**	**8.89**	**<0.001**
